# Body Image Surveys to Address Social Appearance Anxiety in Women at Risk of Eating Psychopathology: An Acceptability, Feasibility and Preliminary Efficacy Study, Using a Wait‐List Randomized Controlled Design

**DOI:** 10.1002/eat.24590

**Published:** 2025-11-05

**Authors:** Emma Giles, Glenn Waller

**Affiliations:** ^1^ School of Psychology University of Sheffield Sheffield UK

**Keywords:** behavioral experiments, body image, cognitive behavioral therapy, eating psychopathology, fear of negative evaluation, social appearance anxiety

## Abstract

**Objective:**

The study investigated the feasibility, acceptability and preliminary efficacy of a body image survey intervention for women at risk of eating psychopathology.

**Method:**

A randomized wait‐list control design was used. The preregistered study (https://doi.org/10.17605/OSF.IO/ZQPEU) had ethical clearance and met recruitment targets. Female participants (aged 18–48 years) with high levels of social appearance anxiety (i.e., deemed at risk of eating pathology) were recruited via advertisement. Thirty‐one participants completed the intervention, with 15 participants randomly allocated to the immediate treatment condition and 16 to the wait‐list condition. The intervention consisted of two thirty‐minute sessions over a week. Self‐report measures were administered every week for 3 weeks and then at the follow‐up 4 weeks later. Acceptability was defined as over 75% of participants completing the study and an average score above 5 on a 7‐point Likert scale assessing acceptability. Feasibility was defined as over 60 participants expressing interest in the study, over 75% of participants consenting to intervention, and over 30 participants recruited for the intervention.

**Results:**

The wait‐list control design met all criteria for feasibility and acceptability. Preliminary outcomes (completer and intention‐to‐treat analyses) suggest body image surveys are effective at reducing social appearance anxiety (*d* = 1.79). While eating psychopathology and body image distress reduced over time, no significant interactions with group were found.

**Discussion:**

While a full trial is needed to add to this evidence, body image surveys appear to be an acceptable and beneficial treatment for people with high levels of social appearance anxiety.

**Summary**

- Body image surveys are deemed acceptable by patients.
- Surveys are strongly effective at reducing social appearance anxiety.
- Body image surveys can be used in clinical practice to target social appearance anxiety.

## 1 Introduction

Eating disorders are severe mental health conditions, associated with significant physical and psychological impairment (Crow et al. 2009; Jenkins et al. 2011; Pohjolainen et al. 2016). They manifest in various forms, all marked by unhealthy eating patterns driven by psychological factors (American Psychiatric Association 2022). It is estimated that over 10% of young women meet criteria for eating disorders and subthreshold eating disorders (Stice et al. 2009; Stice, Becker, and Yokum 2013; Stice, Marti, and Rohde 2013). Eating disorders have a long‐lasting impact on quality of life (Pohjolainen et al. 2016), and the efficacy of treatment is modest in adults (Eddy et al. 2017; Linardon 2018; Monteleone et al. 2022; Treasure et al. 2020; Zipfel et al. 2015). Therefore, prevention and early intervention are viewed as key to improving outcomes and promoting full recovery (Schmidt et al. 2016; Stice, Becker, and Yokum 2013; Stice, Marti, and Rohde 2013; Treasure et al. 2020).

Prevention programs for eating disorders largely focus on reducing body dissatisfaction (Stice, Becker, and Yokum 2013; Stice, Marti, and Rohde 2013), as body image work reduces the likelihood of eating disorder onset (Becker and Stice 2017). Addressing body image concerns is also a central component of cognitive behavioral therapy for eating disorders (CBT‐ED), given the critical role of such concerns in both the development and maintenance of eating disorders (Emanuelli et al. 2012; Fairburn 2008; National Institute of Health and Care Excellence 2020; Treasure et al. 2020; Waller and Beard 2024; Waller et al. 2019). CBT‐ED uses a range of methods to address different manifestations of body image distress (Waller et al. 2019). Some of these methods, such as surveys, could be used in prevention interventions. A survey is a type of behavioral experiment, which is used to test patients' beliefs about what other people think (‘mind‐reading’) (Murray et al. 2022). Surveys involve generating questions with the patient to test the distressing belief (e.g., “They must think I am unattractive”), and then inviting feedback on these questions from people who do not know the person (Murray et al. 2022). For example, a patient might pose a question to others such as: “On a scale of 1‐10, where 1 is very unattractive and 10 is very attractive, how attractive do you think this person's body is?”. The patient would be asked what they predict others would say, which is then tested by comparing the patient's prediction against others' actual feedback. People with high levels of social appearance anxiety are likely to overestimate the likelihood of negative feedback from the survey. As a result, the feedback from others that surveys provide exposes those individuals to corrective information, creating cognitive dissonance (Murray et al. 2022) that is likely to modify their beliefs towards a more realistic position.

### 1.1 Eating Psychopathology and Social Appearance Anxiety

Surveys have the potential to address social appearance anxiety—the anxiety people experience when they fear that other people are judging their appearance, including their body shape, negatively (Hart et al. 2008). It is most common in women (Boursier et al. 2020; Qian et al. 2021), with around 59% of female undergraduates reporting social appearance anxiety in the last year (Gao et al. 2023). Social appearance anxiety is associated with common mental health difficulties (Alcaraz‐Ibanez and Sicilia 2020; Levinson et al. 2013; Pritchard et al. 2021), and is strongly related (*r* = 0.51) to eating psychopathology (Alcaraz‐Ibanez et al. 2023). People who experience high levels of social appearance anxiety are vulnerable to engaging in disordered eating as a safety behavior, which they hope will make their appearance more acceptable to others (Sabiston et al. 2007). Consequently, it has been proposed that social appearance anxiety is a vulnerability factor for eating disorders (Alcaraz‐Ibanez et al. 2023), and is linked with key maintaining behaviors in poor body image (Haase et al. 2007; Sabiston et al. 2007; Waller et al. 2019; White and Warren 2014). Therefore, social appearance anxiety seems to be a key target for eating psychopathology prevention and early intervention.

### 1.2 Use of Surveys for Social Appearance Anxiety

Surveys are commonly used in social phobia. However, they are underutilized in eating disorders, where they can address social appearance anxiety (Murray et al. 2022; Waller et al. 2012). In part, this underutilization is likely to be because surveys are not included in all protocols (e.g., Fairburn 2008). However, it is also important to consider the role of some therapists' reluctance to tolerate unpredictability in therapy for eating disorders, leading to reluctance to employ methods based around behavior change (Turner et al. 2014). Therapists might also be reluctant to use surveys due to fear that they could serve a reassurance‐seeking function and reinforce intolerance of uncertainty among patients with strong investment in their appearance, thus reinforcing social appearance anxiety.

To summarize, surveys are recommended as and when required in some evidence‐based treatments for eating psychopathology, such as CBT‐T (Waller et al. 2019), but there is limited research on their effectiveness (Murray et al. 2022). Therefore, it is clinically necessary to review the efficacy of surveys in isolation, particularly as there is a need for interventions that target social appearance anxiety (Sabiston et al. 2014). At this preliminary stage in the field, it is especially important to begin by evaluating this intervention for people who display high levels of eating and body‐related concerns but do not have a current clinical diagnosis, as reducing social appearance anxiety in this group might help to prevent eating disorders.

Given that this is the first study researching the efficacy of body image surveys in isolation, it will focus on the acceptability and feasibility of such an approach, as well as providing preliminary evidence on their efficacy. A randomized controlled design will be used, with a waiting‐list control condition and follow‐up period, to test the robustness of any evidence of efficacy. Thus, this study will test the potential for further research to demonstrate the impact of surveys more comprehensively. As social appearance anxiety and eating psychopathology are most common in women (Kowalski et al. 2006; Qian et al. 2021), this initial study will focus on adult women, though other group characteristics should be considered in the future (e.g., age, gender, ethnicity).

### 1.3 Aims

1. To investigate whether this wait‐list control design is feasible for researching the efficacy of body image surveys in women at risk of eating psychopathology.
2. To investigate whether a wait‐list control design is an acceptable way of researching the efficacy of a body image survey intervention in such women.
3. To gather pilot evidence about the efficacy of surveys in such women.

### 1.4 Hypotheses

*A wait‐list control design will be feasible in researching a body image survey intervention, according to pre‐determined criteria* (*outlined in Table* *1*).

*A wait‐list control design will be acceptable in researching a body image survey intervention, according to pre‐determined criteria* (*outlined in Table* *1*).

*The survey intervention will result in positive changes in the outcome variables, particularly its central target of social appearance anxiety* (*primary outcome variable*).

**TABLE 1:** Pre‐determined criteria for acceptability and feasibility.

		Red (stop)	Amber (consider amending)	Green (go)
Feasibility	Number of participants expressing interest (completing screening questionnaire)	< 30	30–59	60+
	Percentage of participants who consent to the intervention when offered it	< 50%	50%–75%	> 75%
	Number of participants recruited to complete the intervention	< 20	20–29	30+
Acceptability	Percentage of participants who complete the whole study (including follow‐up measures)	< 50%	50%–75%	> 75%
	Average score on participants' willingness to do the intervention again	< 3	3–5	> 5

## 2 Method

### 2.1 Design

Ethical approval was granted by the University of Sheffield ethics committee. The study was pre‐registered on Open Science Framework (https://doi.org/10.17605/OSF.IO/ZQPEU).

A wait‐list control design was used to address the research aims. Participants with high levels of social appearance anxiety were randomly allocated using an Excel randomizer to either group 1 (immediate intervention) or group 2 (wait list prior to intervention). There was a gap of between 5 and 11 days between time point 1 and time point 2, and between time point 2 and time point 3. This was longer than the aim of 5–8 days, due to the logistics of participants completing both intervention sessions and having time to complete the questionnaires. Time point 4 (Follow‐up) was 4 weeks after time point 3.

### 2.2 Intervention

**FIGURE 1:**
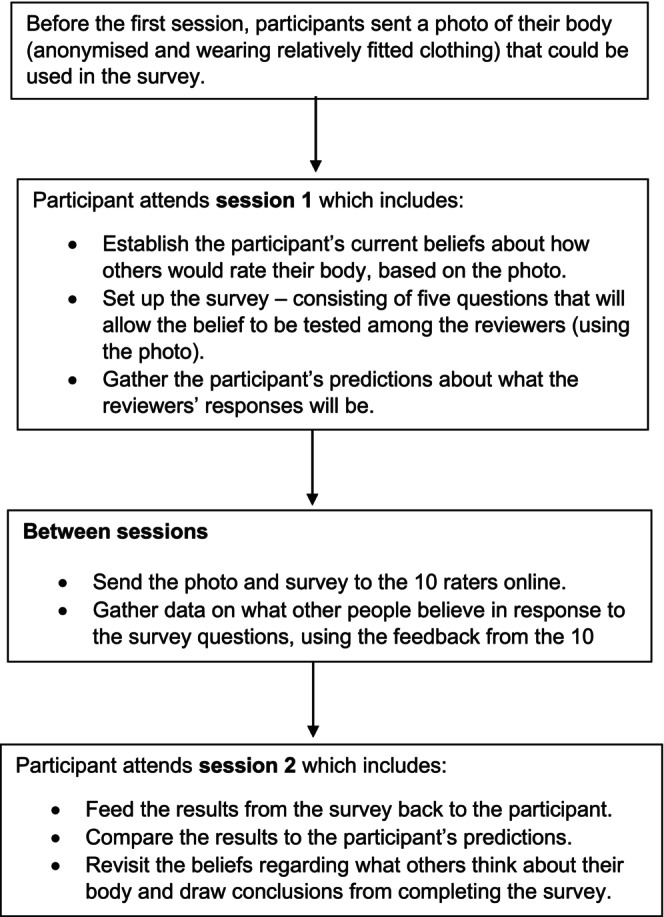
Intervention summary.

The body image surveys were conducted by the lead researcher (Trainee Clinical Psychologist) based on the CBT‐T protocol (Waller et al. 2019), though with a change in the recruitment process for raters (see below). The research supervisor clinically supervised this work to ensure the integrity and quality of the intervention. The intervention consisted of two 30‐min sessions, delivered virtually. The full intervention protocol can be found in the Supporting Information, but a brief description of the intervention is outlined in Figure 1.

### 2.3 Participants

#### 2.3.1 Survey Intervention

Participants were recruited via advertisement within the University of Sheffield and using the University undergraduate research participation system. Participants either received research credits or were entered into a prize draw for two £40 Amazon vouchers in appreciation of their time. At this early stage, participants were told that the study aimed to recruit people with social appearance anxiety, and were asked to complete the screening questionnaire.

Inclusion criteria were cisgender women over the age of 18 (no upper age limit, to ensure feasibility and generalizability) who scored above 40 on the Social Appearance Anxiety Scale (SAAS) in the initial screening questionnaire. While there is no established clinical cut‐off for the SAAS, a score over 40 was deemed appropriate to select participants who had a higher risk of eating and body image psychopathology, as a score of around 30 is average for the population (Levinson and Rodebaugh 2011). Participants were excluded from the study if they reported a current clinically diagnosed eating disorder. People were also excluded if they were unable to speak English fluently.

**FIGURE 2:**
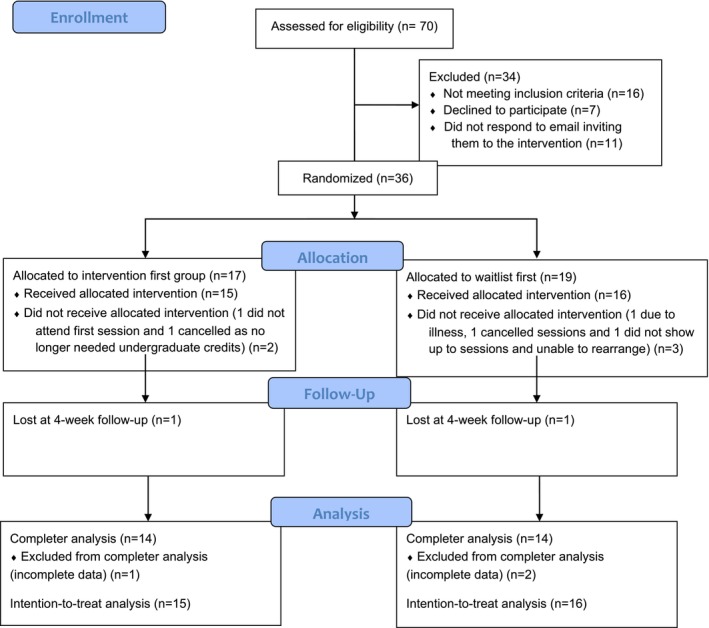
Consort diagram.

A CONSORT diagram is displayed in Figure 2, detailing recruitment and progress. Fifteen people in the ‘active‐wait’ condition and 16 people in the ‘wait‐active’ condition received the full intervention, which is in line with sample size guidance for pilot studies as concluded by Julious (2005). Demographics are displayed in the Results section.

#### 2.3.2 Raters

When this intervention is completed in clinical practice, the rater population is agreed with the client when setting up the behavioral experiment. An example might be using college students on their lunch break to complete anonymised surveys. For this study, the selection of raters was conducted in a more systematic way. Twenty women were recruited via personal and professional contacts to rate the photos. Ten raters were used for each survey, meaning each rater completed between 14 and 16 surveys. Raters were told they were taking part in research around body image, and did not know that their responses would be fed back to participants, as is common in clinical practice. They were required to be 18+ years and fluent in English. The mean age of the raters was 25.1 years (SD = 4.45). Seventeen of the raters were of white ethnicity, and three were of Middle Eastern ethnicity. Raters received a £10 Amazon voucher once they had completed 15 surveys, in appreciation of their time.

### 2.4 Measures

At all four points, participants were sent a Qualtrics link to complete the following measures, each of which was completed at all four time points unless otherwise indicated.

#### 2.4.1 Demographic Questionnaire

At time point 1, demographic data were collected, including age, gender, ethnicity, and self‐reported weight and height.

#### 2.4.2 Social Appearance Anxiety Scale (SAAS; Hart et al. 2008)

The SAAS is a 16‐item self‐report measure, which broadly captures social appearance anxiety rather than focusing narrowly on body shape. Each item is rated on a five‐point Likert type scale (1 not at all; 5 extremely), with higher scores indicating greater social appearance anxiety. An example item is “I am afraid that people find me unattractive”. Scores on the SAAS have been shown to have good internal consistency, convergent validity, and test–retest reliability (Hart et al. 2008). Cronbach's alpha and McDonald's omega scores were above 0.8 for all time points in this study, indicating good internal consistency.

#### 2.4.3 Eating Disorder Examination‐Questionnaire (EDE‐Q7; Grilo et al. 2013)

The EDE‐Q7 consists of seven items of the Eating Disorder Examination‐Questionnaire (Fairburn 2008), which is a widely used questionnaire assessing the frequency of eating disorder symptoms over the past 4 weeks. For this study, the questionnaire was amended to assess the frequency of eating disorder symptoms over the past week. The EDE‐Q7 generates three subscales: Dietary Restraint (e.g., “Have you been consciously trying to limit the amount of food you eat to influence your shape or weight?”), Shape/Weight Overvaluation (e.g., “Has your shape influenced how you think about (judge) yourself as a person?”), and Body Dissatisfaction (e.g., “How dissatisfied have you felt about your shape?”). Items are rated on a 0–6 Likert scale, based on either frequency (e.g., “No days” to “Every day”) or degree (“Not at all” to “Extremely”), with higher scores indicating more eating psychopathology. It has good internal consistency and scales of the EDE‐Q7 are significantly correlated with disordered eating behaviors (Jenkins and Davey 2020). A clinical cut‐off score of 3.64 for the global score has been suggested (Bang et al. 2023). Cronbach's alpha and McDonald's omega scores were above 0.8 for all time points in this study, showing good internal consistency.

#### 2.4.4 Body Shape Questionnaire (BSQ‐8C; Evans and Dolan 1993)

The BSQ‐8C is a short version (eight items) of the Body Shape Questionnaire (Cooper et al. 1987). It measures body satisfaction over the past 4 weeks, with higher scores indicating greater levels of body image dissatisfaction. An example item is “Have you felt excessively large and rounded?”. For this study, the questionnaire was amended to ask participants to consider their body satisfaction over the last week. It has good internal consistency (Kling et al. 2019) and moderate convergent validity (Kling et al. 2019), and can be used in non‐clinical populations (Welch et al. 2012). It also has high sensitivity to change (Pook et al. 2008). A clinical cut‐off score of 26.5 has been suggested (Veisy et al. 2018). Cronbach's alpha and McDonald's omega were above 0.8 for all time points in this study, indicating good internal consistency.

#### 2.4.5 Patient Health Questionnaire‐4 (PHQ‐4; Kroenke et al. 2009)

The PHQ‐4 is a four‐item questionnaire, which combines the brief Patient Health Questionnaire‐2 (PHQ‐2) and Generalized Anxiety Disorder Assessment‐2 (GAD‐2). This creates an anxiety subscale (e.g., “Feeling nervous anxious or on edge”) and a depression subscale (e.g., “Feeling down, depressed, or hopeless”) (Kroenke et al. 2003; Kroenke et al. 2007). Questions are answered on a four‐point Likert scale based on frequency (from “not at all” to “nearly every day”). Higher scores indicate more psychological distress. Although the PHQ‐4 asks people to rate their psychological distress over the last 2 weeks, for this study, participants were asked to rate the questions based on the previous week. Scores of 3 and above for the depression subscale and/or the anxiety subscale suggest probable cases of anxiety or depression. This cut‐off has good sensitivity and specificity (Löwe et al. 2010; Staples et al. 2019). The PHQ‐4 has been found to measure anxiety and depression reliably and validly in the general population (Löwe et al. 2010; Staples et al. 2019), and it is sensitive to treatment changes (Staples et al. 2019). Cronbach's alphas were above 0.8 for all time points in this study for the anxiety and depression subscales, indicating good internal consistency.

#### 2.4.6 Acceptability of the Survey Intervention

Participants were asked whether they would complete the intervention again if offered the opportunity, using a seven‐point Likert scale (1 = totally unwilling; 7 = would be happy to). This was administered at time point 4 only.

### 2.5 Data Analysis

To analyze acceptability and feasibility (Hypotheses 1 and 2), performance was monitored against the pre‐defined stop‐go criteria provided in Table 1. As stop‐go criteria are different across papers and for specific interventions, we determined our criteria by what we saw as appropriate to support the use of such a technique in routine clinical practice. That included: a large majority of participants who would agree to try the method (75%); the majority completing the method (> 50%); the great majority also completing the outcome measures (> 75%); and a strong level of willingness to undertake further survey work (> 5/7). It is not suggested that these are cut‐offs that should be applied universally, as they should be set according to the specific clinical intervention. However, the application of such criteria is a valuable element in ensuring the viability and replicability of any intervention method.

SPSS (version 29) was used to analyze the questionnaire data (Hypothesis 3). For the completer analysis, a set of 2 × 4 repeated measures ANOVAs were used to compare outcomes between groups across the four time points. Alpha level was set at 0.05. Effect sizes were reported as partial eta^2^, where a score of ≥ 0.14 indicates a large effect, 0.06–0.13 indicates a medium effect, and 0.01–0.05 indicates a small effect (Cohen 1992). Independent sample *t*‐tests were used to interpret the interaction of time by group, reporting Cohen's *d* (*d* = (Mean1—Mean2)/Pooled Standard Deviation) as the effect size. For the intention‐to‐treat analysis, generalized linear mixed models were used.

Normality testing was completed (see Supporting Information), and showed broadly normal distributions (particularly for the SAAS). As ANOVAs can cope robustly with skewness and kurtosis (Box et al. 1978), these findings indicated that parametric analysis was suitable. Mauchley's test of sphericity was calculated, and if this was significant, the Greenhouse–Geisser correction was used. Cronbach's alpha and McDonald's omega (where there were more than two items in the scale) were calculated at each time point, to ensure internal consistency of the scales.

## 3 Results

### 3.1 Feasibility and Acceptability (Aims 1 and 2)

All the feasibility criteria were met (see Figure 2). Seventy potential participants expressed interest in the study, which was higher than the target of 60 (Table 1). Of those who were eligible, 36 out of 43 (83.72%) consented to take part in the intervention when offered, which was higher than the target of 30 (Table 1).

All the acceptability criteria were met (see Figure 2). Twenty‐eight participants (77.8%) completed the whole study, including follow‐up measures, which was higher than the 75% target (Table 1). The average acceptability score was 5.72, which was higher than the target of 5.0 (Table 1).

### 3.2 Sample Characteristics

Table 2 shows the characteristics of the completing participants in the two groups.

**TABLE 2:** Demographics.

	Active‐wait (*n* = 14)	Wait‐active (*n* = 14)
Age mean (SD)	21.07 (4.81)	23.79 (9.06)
	Range (18–36)	Range (18–48)
Ethnicity	9 White	10 White
	2 Asian	4 Asian
	2 Mixed Ethnicity	
	1 Middle eastern	
BMI mean (SD)	23.84 (5.27)	23.61 (6.16)
	(Range = 17.4–36.8)	(Range = 17.7–35.2)

### 3.3 Preliminary Efficacy of the Survey Intervention (Aim 3)

Table 3 (repeated measures ANOVA) and Table 4 (Generalized Linear Mixed Model) display means/estimated means and standard deviations/standard errors for all the measures across time for each group.

**TABLE 3:** Means and standard deviations and repeated measures ANOVAs.

		TP 1 mean (SD)	TP 2 mean (SD)	TP 3 mean (SD)	TP 4 mean (SD)	Time			Group			Group × time		
						*F* (3,28)	*p*	Partial eta^2^	*F* (1,26)	*p*	Partial eta^2^	*F* (3,28)	*p*	Partial eta^2^
SAAS	Active‐wait	56 (8.88)	43.5 (7.49)	45.57 (8.92)	43.07 (8.39)	**33.79**	**< 0.001**	**0.57**	1.74	0.198	0.06	**13.79**	**< 0.001**	**0.34**
	Wait‐Active	58.29 (10.16)	59.43 (10.08)	43.64 (11.02)	44.29 (14.20)									
EDEQ‐7 global	Active‐wait	3.5 (1.13)	2.92 (1.30)	2.87 (1.39)	2.54 (1.19)	**15.21**	**< 0.001**	**0.37**	0.99	0.755	0.00	1.98	0.142	0.07
	Wait‐active	3.83 (1.44)	3.51 (1.52)	2.66 (1.57)	2.44 (1.73)									
EDEQ‐7 DT	Active‐wait	2.36 (1.71)	2.02 (1.76)	2.17 (2.13)	1.40 (1.18)	**4.16**	0.**010**	**0.14**	0.48	0.496	0.02	0.33	0.807	0.01
	Wait‐active	2.74 (1.96)	2.64 (2.01)	2.36 (1.94)	2.0 (2.22)									
EDEQ‐7 SWO	Active‐wait	4.36 (1.12)	3.64 (1.33)	2.54 (1.45)	3.43 (1.49)	**18.21**	**< 0.001**	**0.41**	0.00	0.951	0.00	2.20	0.108	0.08
	Wait‐active	4.61 (1.34)	4.00 (1.47)	2.71 (1.24)	2.54 (1.93)									
EDEQ‐7 BD	Active‐wait	4.36 (1.18)	3.53 (1.31)	3.46 (1.31)	3.36 (1.38)	**14.41**	**< 0.001**	**0.35**	0.02	0.985	0.00	**3.17**	0.**04**	**0.12**
	Wait‐active	4.68 (1.41)	4.32 (1.61)	2.96 (1.78)	3.00 (1.63)									
BSQ‐8c	Active‐wait	30.14 (9.26)	25.71 (8.75)	25.29 (8.01)	24.79 (8.32)	**16.09**	**< 0.001**	**0.38**	0.00	0.960	0.00	**3.30**	0.**026**	**0.11**
	Wait‐active	30.0 (10.06)	28.71 (8.88)	25.43 (8.61)	21.14 (9.93)									
PHQ‐4 A	Active‐wait	3.43 (1.79)	2.57 (1.34)	2.14 (1.46)	2.43 (1.65)	1.84	0.148	0.07	0.01	0.462	0.00	1.88	0.14	0.07
	Wait‐active	2.64 (1.65)	2.53 (1.55)	2.71 (2.09)	2.57 (2.21)									
PHQ‐4 D	Active‐wait	1.57 (1.34)	1.29 (0.83)	0.79 (0.70)	1.43 (1.40)	1.05	0.378	0.04	**4.52**	0.**043**	**0.15**	0.50	0.616	0.02
	Wait‐active	2.50 (1.61)	2.13 (1.96)	2.29 (2.27)	2.36 (2.21)									

*Note*: Bold—*p* < 0.05.

Abbreviations: A, anxiety; BD, body dissatisfaction; BSQ‐8c, body shape questionnaire‐8c; D, depression.; DT, drive for thinness; EDEQ‐7, eating disorder examination questionnaire‐7; PHQ‐4, patient health questionnaire 4; SAAS, social appearance anxiety scale; SWO, shape and weight overevaluation; TP, time point.

**TABLE 4:** Generalized linear mixed models, showing intention‐to‐treat analyses.

		TP 1 estimated mean (SE)	TP 2 estimated mean (SE)	TP 3 estimated mean (SE)	TP 4 estimated mean (SE)	Time	Group	Group × time
SAAS	Active‐wait	55.47 (2.41)	43.27 (2.34)	45.40 (2.59)	43.07 (3.16)	***F* (3,114) = 12.11, *p* < 0.001**	*F* (1,114) = 3.67, *p* = 0.058	***F* (3,114) = 4.71, *p* = 0.004**
	Wait‐active	58.00 (2.34)	57.44 (2.27)	42.69 (2.51)	43.2 (3.06)			
EDEQ‐7 global	Active‐wait	3.39 (0.33)	2.84 (0.35)	2.77 (0.38)	2.54 (0.40)	***F* (3,114) = 4.22, *p* = 0.007**	*F* (1,114) = 0.35, *p* = 0.56	*F* (3,114) = 0.64, *p* = 0.59
	Wait‐active	3.80 (0.32)	3.42 (0.34)	2.56 (0.37)	2.35 (0.38)			
EDEQ‐7 DT	Active‐wait	2.20 (0.47)	1.89 (0.48)	2.02 (0.52)	1.41 (0.48)	*F* (3,114) = 1.10, *p* = 0.354	*F* (1,114) = 1.83, *p* = 0.18	*F* (3,114) = 0.06, *p* = 0.983
	Wait‐active	2.73 (0.45)	2.50 (0.47)	2.25 (0.50)	1.87 (0.46)			
EDEQ‐7 SWO	active‐wait	4.23 (0.33)	3.53 (0.36)	2.43 (0.34)	3.43 (0.45)	***F* (3,114) = 11.65, *p* < 0.001**	*F* (1,114) = 0.02, *p* = 0.90	*F* (3,114) = 1.09, *p* = 0.36
	Wait‐active	4.53 (0.32)	3.88 (0.35)	2.63 (0.33)	2.47 (0.44)			
EDEQ‐7 BD	Active‐wait	4.33 (0.32)	3.57 (0.36)	3.30 (0.41)	3.36 (0.40)	***F* (3,114) = 7.55, *p* < 0.001**	*F* (1,114) = 0.08, *p* = 0.78	*F* (3,114) = 1.29, *p* = 0.28
	Wait‐active	4.68 (0.31)	4.34 (0.35)	2.84 (0.39)	2.97 (0.38)			
BSQ‐8c	Active‐wait	29.07 (2.45)	24.73 (2.28)	24.27 (2.20)	24.79 (2.42)	*F* (3,114) = 3.05, *p* = 0.31	*F* (1,114) = 0.11, *p* = 0.75	*F* (3,114) = 1.15, *p* = 0.33
	Wait‐active	30.06 (2.41)	29.25 (2.21)	24.94 (2.13)	20.73 (2.34)			
PHQ‐4 A	Active‐wait	3.60 (0.45)	2.80 (0.43)	2.27 (0.46)	2.43 (0.51)	*F* (3,114) = 0.71, *p* = 0.55	*F* (1,114) = 0.39, *p* = 0.53	*F* (3,114) = 1.02, *p* = 0.39
	Wait‐active	2.5 (0.44)	2.53 (0.42)	2.63 (0.44)	2.6 (0.49)			
PHQ‐4 D	Active‐wait	1.8 (0.49)	1.53 (0.45)	1.00 (0.48)	1.43 (0.49)	*F* (3,114) = 0.24, *p* = 0.87	***F* (1,114) = 7.57, *p* = 0.007**	*F* (3,114) = 0.33, *p* = 0.81
	Wait‐active	2.31 (0.39)	2.31 (0.43)	2.38 (0.47)	2.27 (0.47)			

*Note*: Bold—*p* < 0.05.

Abbreviations: A, anxiety; BD, body dissatisfaction; BSQ‐8c, body shape questionnaire‐8c; D, depression; DT, drive for thinness; EDEQ‐7, eating disorder examination questionnaire‐7; PHQ‐4, patient health questionnaire‐4; SAAS, social appearance anxiety scale; SWO, shape and weight overevaluation; TP, time point.

#### 3.3.1 Main Effects

For the *completer analysis*, repeated measures ANOVAs showed significant main effects of Time for social appearance anxiety, eating psychopathology and body image, where scores improved with very large effect sizes (Table 3). For the *intention‐to‐treat* analysis, generalized linear mixed models showed significant main effects of Time for social appearance anxiety and eating psychopathology (global, shape and weight overevaluation, body dissatisfaction), showing positive benefits (Table 4).

There were no significant effects of Group for any variable apart from PHQ depression. In that case, the waiting list group scored higher than the initially active group on both the completer and intention‐to‐treat analyses (Tables 3 and 4).

#### 3.3.2 Group × Time Interactions

**FIGURE 3:**
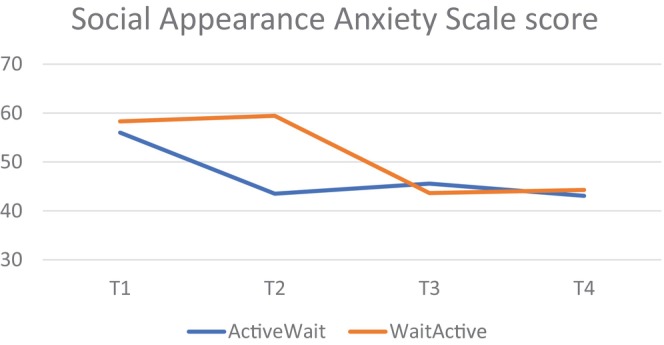
Social appearance anxiety—time × group interaction (completer analysis—repeated measures ANOVA).

**FIGURE 4:**
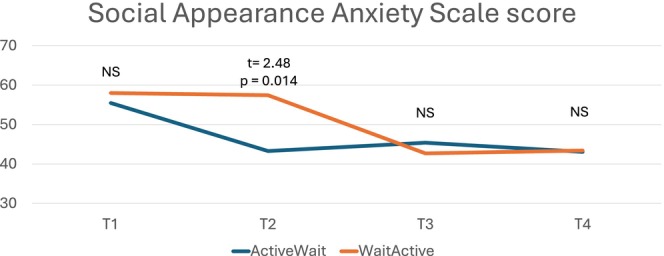
Social appearance anxiety—time × group interaction (estimated means from generalized linear mixed models, with independent t‐tests to compare time points).

In the *completer analysis*, there were three significant Group × Time interactions, with effect sizes shown in Table 3. However, the independent samples t‐tests showed that there was only one measure where there was a significant difference between groups at the critical point of Time 2—on the SAAS (*t* = 4.706, *p* < 0.001, *d* = −1.749). The other two significant interactions (BSQ‐8 and EDEQ‐7 BD) did not show significant *t*‐tests at this time point (*t* = 1.05 and *t* = 1.42, respectively). This timepoint is critical because it is where the active‐wait group had received the intervention and the wait‐active group had not. The effect size of the difference was very large (see Table 3 and Figure 3). This finding was replicated in the *intention‐to‐treat* analysis, where SAAS was the only significant interaction (Table 4 and Figure 4).

## 4 Discussion

This is the first study researching the efficacy of body image surveys in isolation. Therefore, the aim was to investigate whether this wait‐list control design is a feasible and acceptable way for researching the efficacy of a body image survey intervention in women at risk of eating psychopathology. The study also gathered pilot evidence about the efficacy of that survey intervention in such women.

The findings demonstrated the feasibility and acceptability of the use of surveys, supporting Hypotheses 1 and 2. Hypothesis 3 was partially supported. In both completer and intention‐to‐treat analyses, the intervention was effective in significantly reducing the primary outcome variable of social appearance anxiety, with a very large effect size. However, although there were significant reductions over time in mean scores for eating psychopathology and body image distress, no robust time × group interactions were found. The survey intervention was not effective at reducing depression and anxiety.

### 4.1 Relationship of Findings to the Wider Literature

Social anxiety and eating psychopathology are highly comorbid (Levinson and Rodebaugh 2012; Kerr‐Gaffney et al. 2018), with high social anxiety leading to more severe eating psychopathology (Kerr‐Gaffney et al. 2018). Social appearance anxiety and fear of negative evaluation are vulnerabilities for both social anxiety and eating disorder symptoms (Levinson and Rodebaugh 2012). It is, therefore, important to address fear of negative evaluation in body image and eating disorder interventions (DeBoer et al. 2013; Trompeter et al. 2019).

This study adds to the evidence about how social appearance anxiety, a type of body image distress based in fear of negative evaluation of appearance, can be treated. A meta‐analytic review suggests that most brief stand‐alone interventions for body image lead to small–medium effect sizes (Alleva et al. 2015). However, studies using stand‐alone behavioral experiments were not identified in that review or in more recent papers that use interventions such as acceptance and commitment therapy (Li et al. 2025; Mirzaei et al. 2023; Srichan et al. 2023), CBT (Dogaheh et al. 2011; Mirzaei et al. 2023) or psychoeducation (Ghosh et al. 2023) for fear of negative evaluation. In short, there is insufficient research into focused interventions for fear of negative evaluation and social appearance anxiety.

Considering the nature of this gap in our understanding, it is important to remember that behavioral experiments are among the most powerful stand‐alone change methods (Bennett‐Levy et al. 2004), leading to more behavioral and belief change than thought records (Bennett‐Levy 2003). Therefore, it is consistent that, in this study, body image surveys should have produced very large reductions in social appearance anxiety.

Whilst social appearance anxiety was significantly reduced with the intervention, significant interaction effects were not found for the secondary variables. The lack of secondary effects might be due to the intervention being limited to two sessions, when it is possible that more sessions would be needed for the optimal dose to address this form of eating disorder psychopathology (Robinson et al. 2020; Rose and Waller 2017). Furthermore, body image has many maintaining factors, so other outcomes (e.g., change in body image, mood and general eating pathology) are likely to depend on the use of a wider range of body image interventions over a longer period of time (Waller et al. 2019).

### 4.2 Research Implications and Limitations

Whilst this study has strengths, such as using a randomized controlled design and considering multiple outcomes of the intervention, it has a number of limitations. First, acceptability was measured in a limited way (completion of the task and a Likert scale). Future research should consider assessing acceptability more broadly by qualitatively exploring participants' experiences of the intervention and the acceptability of the measures used. Second, it is possible that participants used additional support during the experiment, which should be assessed and controlled for. Third, this study also had a relatively small sample size, limiting power at post hoc testing. Therefore, as this design met pre‐determined acceptability and feasibility criteria, a full trial with adequate power is needed, based on the effect sizes for different outcomes demonstrated in this study. Such a full trial should also strive to regularize gaps between the administration of measures. Furthermore, such research should establish the effectiveness of body image surveys in other populations (e.g., men, gender non‐conforming individuals), given that the role of social appearance anxiety in the development of eating psychopathology and other mental health conditions might differ across gender identities (Haase et al. 2002; Klemmer et al. 2021; Li et al. 2024; Turel et al. 2018).

Returning to a point raised in the Introduction, it is possible to hypothesize that surveys might increase reassurance‐seeking in the longer term, and thus social appearance anxiety. While CBT protocols using surveys have shown sustained improvements (e.g., Pellizzer et al. 2019), it is possible that those studies did not monitor psychological change over a long enough period to see negative outcomes (enhanced reassurance‐seeking resulting in increases in intolerance of uncertainty, and hence a resurgence of social appearance anxiety). Larger studies than this one are needed, with longer‐term follow‐up periods, specifically attending to this potential mechanism behind body image following therapy.

Finally, validation of these findings in clinical groups is necessary, in keeping with the need for evaluation of focused interventions for different presentations of eating disorders (Davey et al. 2023). This is particularly important, as social appearance anxiety is a common maintaining factor in eating psychopathology (Alcaraz‐Ibáñez et al., Alcaraz‐Ibanez et al. 2023; Kowalski et al. 2006; Lanfranchi et al. 2015; Sabiston et al. 2014). Such research should compare outcomes among individuals with different weight/BMI status, to determine the generalizability of the effect. In all such groups, it would also be helpful to evaluate the effectiveness of different types of surveys (e.g., interpersonal and intrapersonal) and repeated surveys (Murray et al. 2022; Waller et al. 2019).

### 4.3 Clinical Implications

As this study found that individuals view surveys as highly acceptable and effective for reducing social appearance anxiety, body image surveys might be effective as a preventative intervention for eating psychopathology. Such an intervention would be in keeping with fear of negative evaluation being an important target in prevention programs (DeBoer et al. 2013) and social appearance anxiety being a known vulnerability factor for eating psychopathology (Alcaraz‐Ibanez et al. 2023). Such an approach links to the importance of early intervention in eating psychopathology to promote full recovery (Schmidt et al. 2016; Treasure et al. 2020). Considering the possibilities raised in the Introduction, it should be noted that these positive outcomes are not consistent with clinical concerns that undertaking surveys serves any reassurance‐seeking function for the individual who has a strong investment in their appearance. However, further studies are needed to address this concern directly, now that the viability and overall effectiveness of surveys have been given empirical support. Additionally, while therapists are often fearful that the distressing belief will be confirmed by the survey, we stress that the outcome rarely comes close to realizing that fear. The degree of error in patients' prediction about what others will say is usually very large, meaning that even with variability in the raters' scores (e.g., mean fatness rating = 3, range = 1–5), it rarely results in even individual raters appearing to support the patient's predictions based on mind‐reading (e.g., mean fatness rating = 8, range = 7–10). Of course, were it the case that an individual rater came close to confirming the patient's belief (e.g., rating = 7), this can be attended to by focusing on the average rater scores rather than individual rater scores, and through thorough debriefing, as would be done in other behavioral experiments (Kennerley et al. 2017).

Finally, as social appearance anxiety is common in the general population (Kowalski et al. 2006; Sabiston et al. 2014), body image surveys should also be considered as a possible tool to promote positive body image in the non‐clinical population. A single‐session intervention approach could be helpful for this (e.g., Schleider and Beidas 2022; Schleider and Weisz 2017).

### 4.4 Conclusions

This is the first study researching the efficacy of body image surveys for social appearance anxiety. The wait‐list randomized controlled design was feasible and acceptable, and there is preliminary evidence that such surveys are effective at reducing social appearance anxiety. While a full trial is needed to add to this evidence, these findings suggest a promising future line of clinical research.

## Author Contributions

**Emma Giles:** conceptualization, investigation, writing – original draft, formal analysis, project administration. **Glenn Waller:** conceptualization, writing – review and editing, supervision, formal analysis.

## Conflicts of Interest

The authors declare no conflicts of interest.

## Supporting information


**Data S1:** Supporting Information.

**Data S2:** Supporting Information.

## Data Availability

The data that support the findings of this study are available from the corresponding author upon reasonable request.
