# Supplementary material for: Body Image Surveys to Address Social Appearance Anxiety in Women at Risk of Eating Psychopathology: An Acceptability, Feasibility and Preliminary Efficacy Study, Using a Wait‐List Randomized Controlled Design
Source: Int J Eat Disord. 2025 Nov 5;59(2):371–83. doi: 10.1002/eat.24590 (PMC12884258; doi:10.1002/eat.24590)
Supplement: Supplementary file 1 — Data S1: Supporting Information. [file EAT-59-371-s002.docx]

**Intervention Protocol**

**The intervention**

The body image surveys will be conducted by the lead researcher (Trainee Clinical Psychologist) based on the CBT-T protocol (Waller et al., 2019). The intervention will consist of two 30-minute sessions, delivered virtually. Professor Glenn Waller will clinically supervise this work to ensure integrity and quality of the intervention. Participants will be asked to send an anonymized photograph of their body before the first session (that they feel represents a key issue in their body image e.g. size, disproportionate parts, unattractive aspects). The session plans are outlined below:

Session 1  - throughout the first session I will take a Socratic approach – assuming that patients might be right before seeking evidence to challenge that belief – so that the patient doesn’t feel their belief is being discounted

1. Introduce myself and the purpose of the session – *“I am Emma, a trainee psychologist from Sheffield. Thank you for consenting to participate in this research and thank you for sending me the photo. Today we have 30 minutes together to develop the survey, which will involve thinking about what beliefs we want to test and what questions we can ask to test these beliefs. After we have done this, I will then collect responses to the questions we develop from around 10 women who are neutral and who will think they are taking part in some research about perceptions of female bodies. This is to ensure that the responses you get are honest. We will then meet again next week for me to feed these results back to you. Does that sound okay? Have you got any questions?”*
2. Establish current beliefs about what other people think about their bodies - *“When I show people this photo what do you fear people will think? What do you worry other people think about your body?”*
3. Set up a questionnaire of 5 questions that will allow the belief to be tested. The first question we will ask in the all the surveys will be “what are the three first things you noticed about this person’s body?” We will aim for all other questions to be dimensional e.g. On a scale of 1 to 10….., how….“*So let’s write down some questions that you could ask to find out if you are right or wrong. How about….”*
4. We will then start to consider what the participant predicts the responses will be to each question (e.g. average score will be 8) “*Okay I have written down those questions, based on this photo. So what do you think people will say when I ask them? What percentage of people do you think will say that? What do you think the average score will be? Etc”*
5. *C*onclude the session *“Okay… so I will go and gather responses to the questions we have developed and then I will meet you next week to feed back the results. In the meantime, if you need support then I recommend the BEAT website or if you feel that further counselling would be helpful, you can self-refer to IAPT or to the University Health Service.”*

Between sessions

1. Gather the data on what other people believe from raters who I have recruited 2 weeks before the intervention. Each survey will take around 5 minutes to complete and I will ask people to do about 10-15 surveys at a time. Raters will be given a £10 amazon voucher to thank them for their time.

Session 2

1. Introduction to the session “*Hello – it’s nice to see you again. So last session we developed some questions and you made some predictions of what you thought people would say. We have 30 minutes today to go through the responses together.”*
2. Go over the predictions before the feedback *“ you predicted that…. so shall we see if you were right?”*
3. Feedback the responses one by one “So *for the first question the first rater said…. “*
4. I will share my screen so that the participant can see the word document and I will add the responses one by one.

| Question | My prediction (what I think people will say) | Responses to survey |
| --- | --- | --- |
|  |  | 1.  2.  3. |
|  |  |  |
|  |  |  |

1. Compare the results to the patient’s ratings. “So… *you predicted that…. However…… what do you conclude from that?”*
2. Summarise and reflect on the session. “*Can you summarise the results overall and how they contrast with the predictions you made? What do you make of that? What does that say about your beliefs about what other think about your appearance?*
3. Ending the session “*I will email you a copy of this table so that you have got the results. This is a technique that you can continue to use to test beliefs, if you wish. Thank you for completing the intervention – it has been good to meet with you. Following this session, I will send you a link to some questionnaires, and then I will send you a similar link in 4 weeks – it is really important that you complete these questionnaires so that we can evaluate the intervention. If you need any further support I recommend the BEAT website, or if you feel that further counselling would be helpful, you can self-refer to IAPT.”*
