# Supplementary material for: Body Image Surveys to Address Social Appearance Anxiety in Women at Risk of Eating Psychopathology: An Acceptability, Feasibility and Preliminary Efficacy Study, Using a Wait‐List Randomized Controlled Design
Source: Int J Eat Disord. 2025 Nov 5;59(2):371–83. doi: 10.1002/eat.24590 (PMC12884258; doi:10.1002/eat.24590)

**Supplementary material: Normality tests for ANOVAs by dependent variables**

**SAAS**

***Skewness and Kurtosis***

|  | **Wait-active** | | **Active Wait** | |
| --- | --- | --- | --- | --- |
|  | **Skewness** | **Kurtosis** | **Skewness** | **Kurtosis** |
| **Time point 1** | -0.052 | -1.138 | .597 | 1.154 |
| **Time point 2** | -0.392 | 0.191 | .417 | 0.09 |
| **Time point 3** | 0.754 | -0.493 | 0.129 | 0.116 |
| **Time point 4** | 0.679 | -0.418 | -0.341 | -0.642 |

***Shapiro-Wilk Test***

|  | **Wait-active** | | **Active Wait** | |
| --- | --- | --- | --- | --- |
|  | **Statistic** | **Significance** | **Statistic** | **Significance** |
| **Time point 1** | 0.955 | 0.637 | 0.196 | 0.107 |
| **Time point 2** | 0.982 | 0.984 | 0.966 | 0.824 |
| **Time point 3** | 0.900 | 0.113 | 0.991 | 1 |
| **Time point 4** | 0.917 | 0.2 | 0.953 | 0.601 |


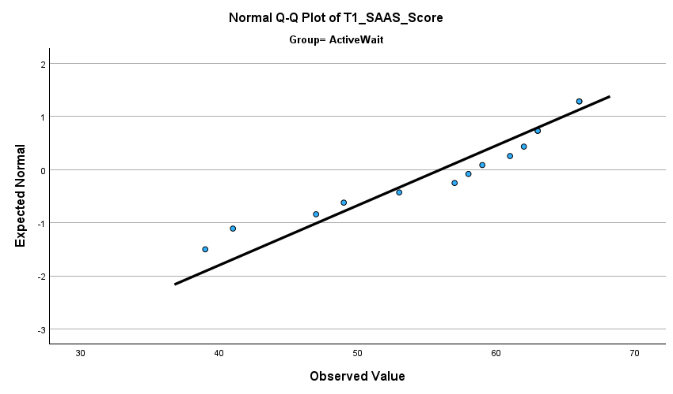

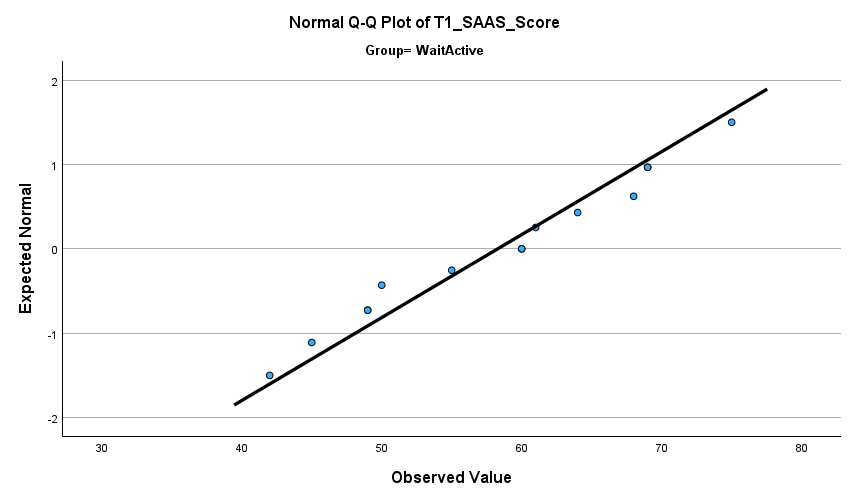


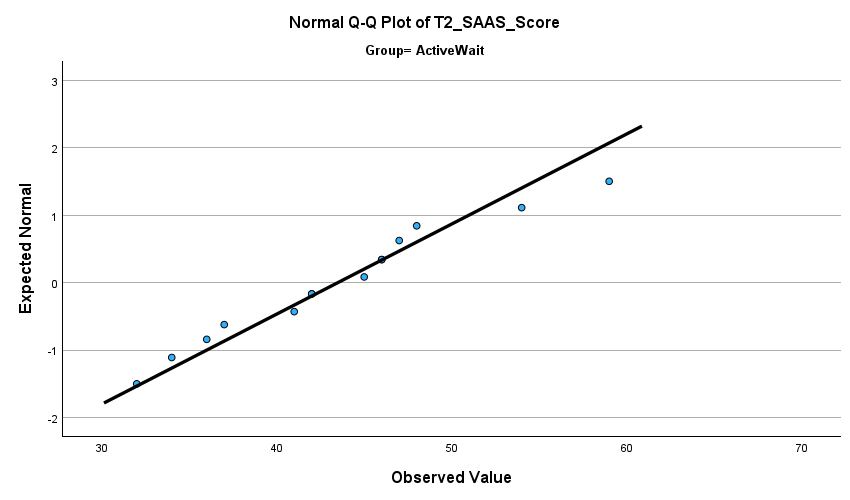

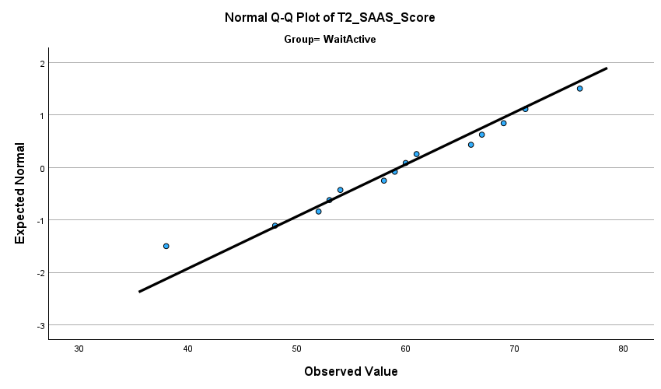


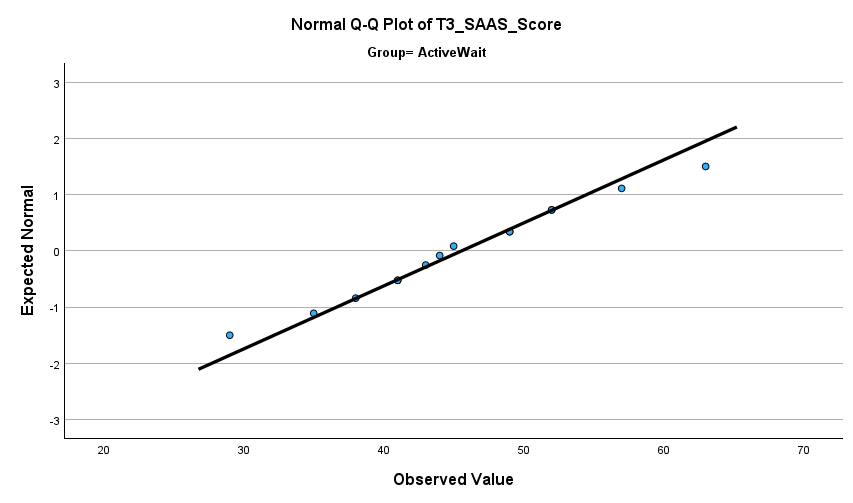

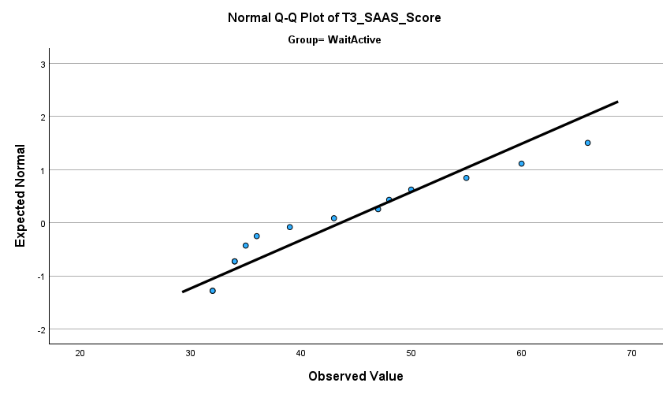


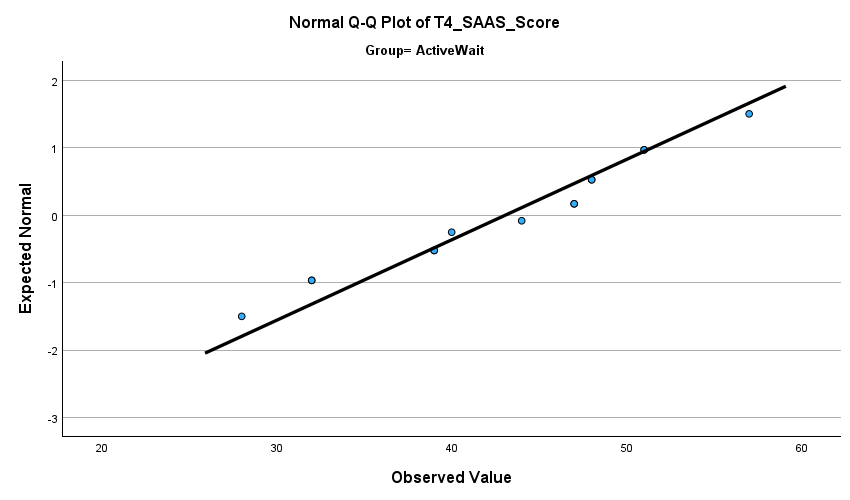

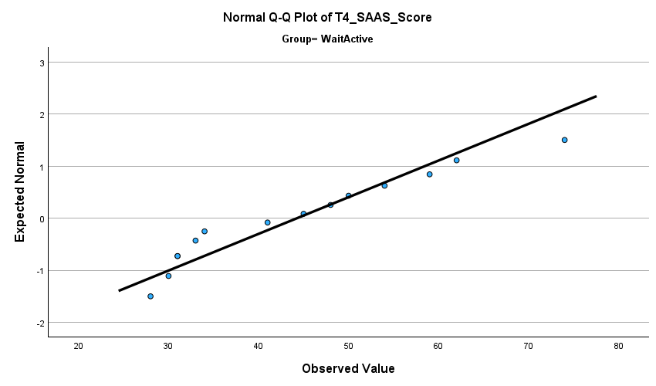


**BSQ**

***Skewness and Kurtosis***

|  | **Wait-active** | | **Active Wait** | |
| --- | --- | --- | --- | --- |
|  | **Skewness** | **Kurtosis** | **Skewness** | **Kurtosis** |
| **Time point 1** | -0.163 | -1.281 | 0.063 | -1.001 |
| **Time point 2** | -0.009 | -0.778 | .499 | -0.483 |
| **Time point 3** | 0.377 | -0.976 | 0.467 | -0.698 |
| **Time point 4** | 1.120 | 0.599 | 0.07 | -1.668 |

***Shapiro-Wilk Test***

|  | **Wait-active** | | **Active Wait** | |
| --- | --- | --- | --- | --- |
|  | **Statistic** | **Significance** | **Statistic** | **Significance** |
| **Time point 1** | 0.925 | 0.260 | 0.955 | 0.638 |
| **Time point 2** | 0.957 | 0.673 | 0.926 | 0.270 |
| **Time point 3** | 0.948 | 0.532 | 0.943 | 0.452 |
| **Time point 4** | 0.868 | 0.04 | 0.899 | 0.109 |


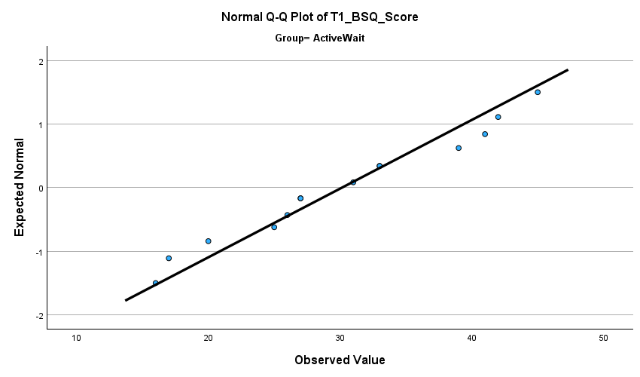

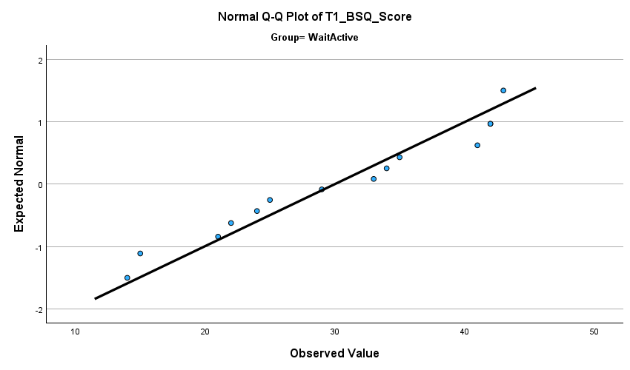


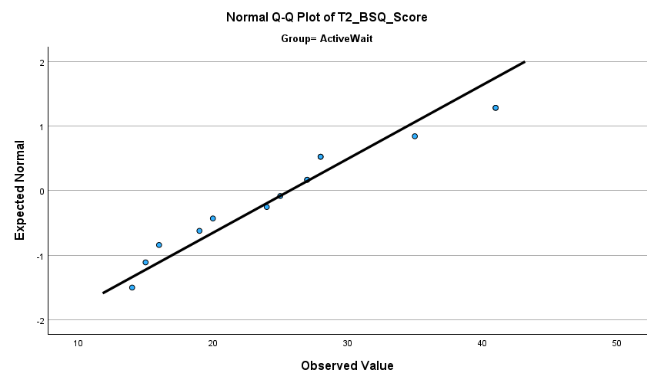

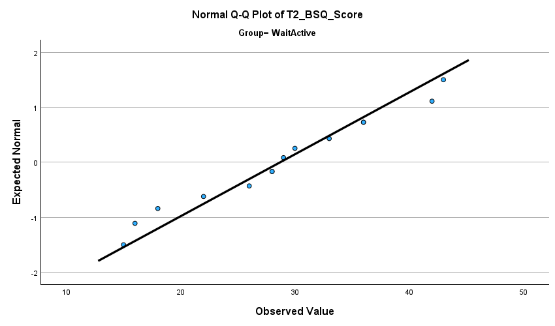


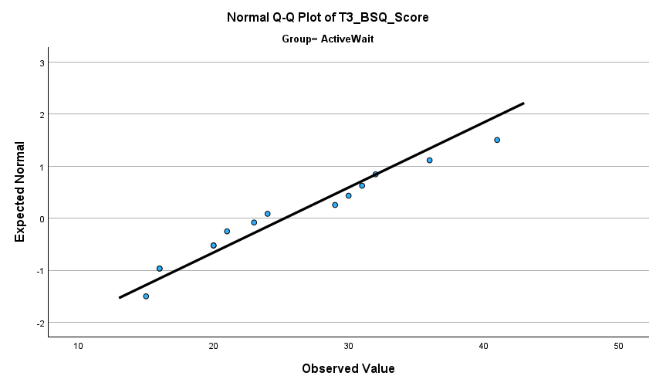

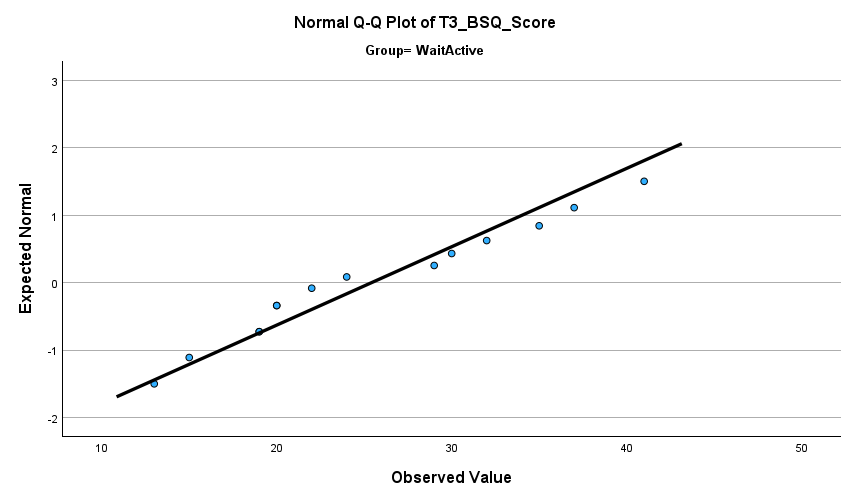


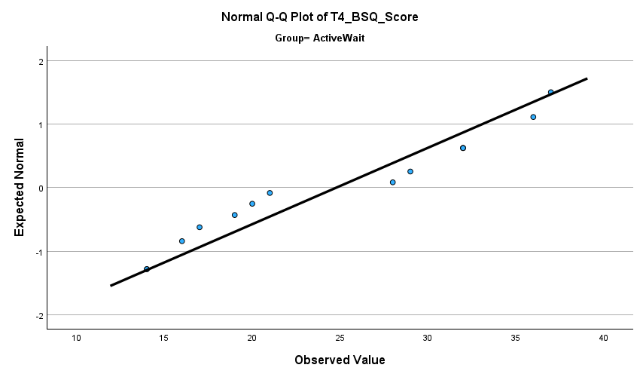

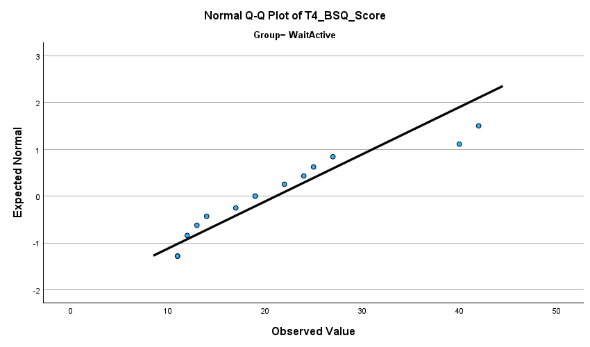


**EDEQ-Global**

***Skewness and Kurtosis***

|  | **Wait-active** | | **Active Wait** | |
| --- | --- | --- | --- | --- |
|  | **Skewness** | **Kurtosis** | **Skewness** | **Kurtosis** |
| **Time point 1** | 0.247 | -1.404 | 0.458 | -0.129 |
| **Time point 2** | 0.185 | -1.028 | 0.861 | 0.883 |
| **Time point 3** | 0.689 | 0.123 | 0.721 | 0.624 |
| **Time point 4** | 1.102 | 0.250 | 0.011 | -0.069 |

***Shapiro-Wilk Test***

|  | **Wait-active** | | **Active Wait** | |
| --- | --- | --- | --- | --- |
|  | **Statistic** | **Significance** | **Statistic** | **Significance** |
| **Time point 1** | 0.918 | 0.203 | 0.950 | 0.557 |
| **Time point 2** | 0.959 | 0.704 | 0.931 | 0.316 |
| **Time point 3** | 0.936 | 0.368 | 0.959 | 0.702 |
| **Time point 4** | 0.870 | 0.042 | 0.987 | 0.997 |


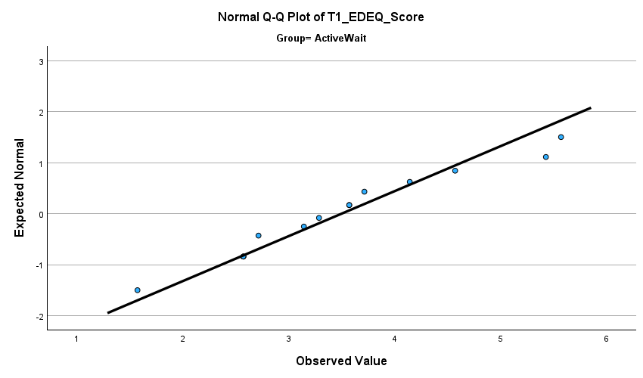

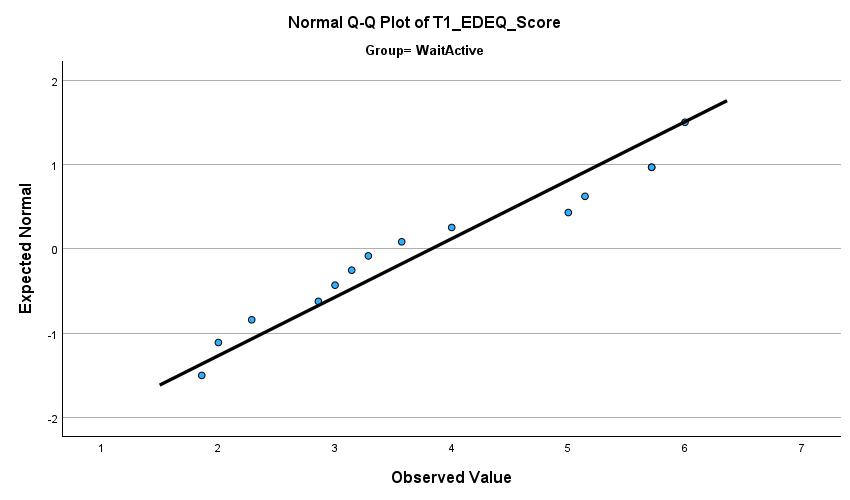


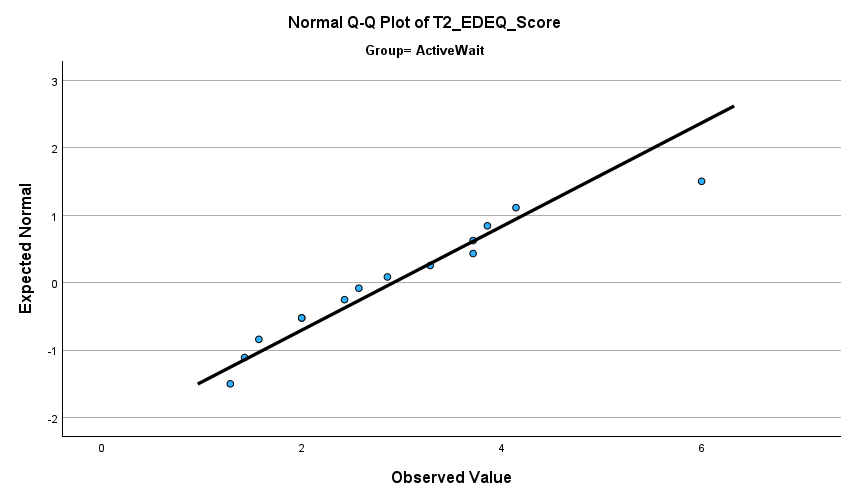

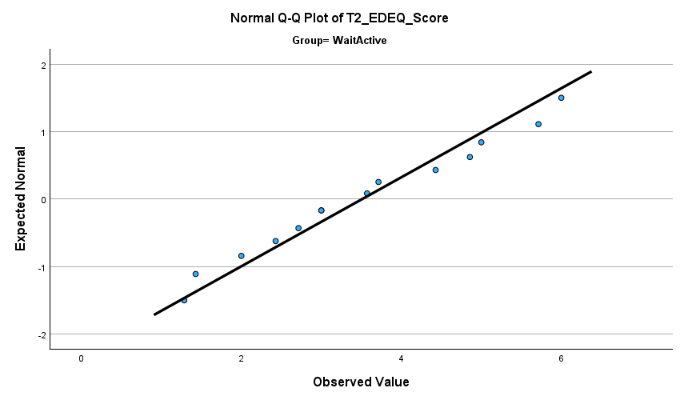


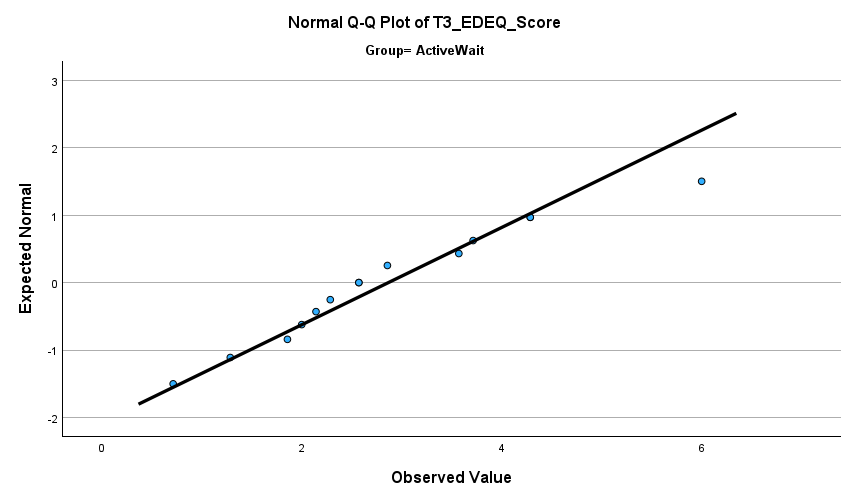

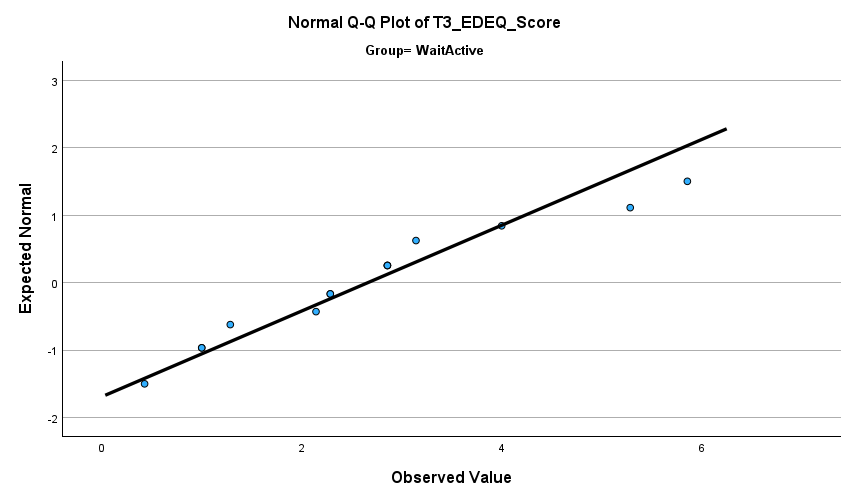


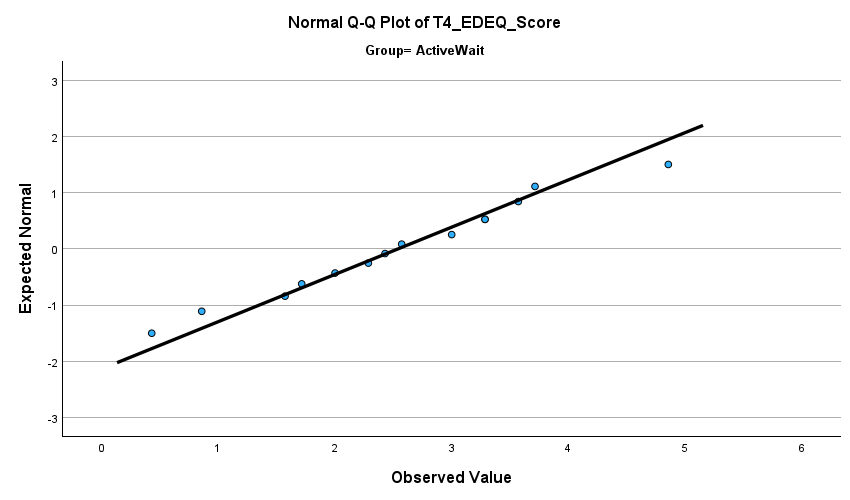

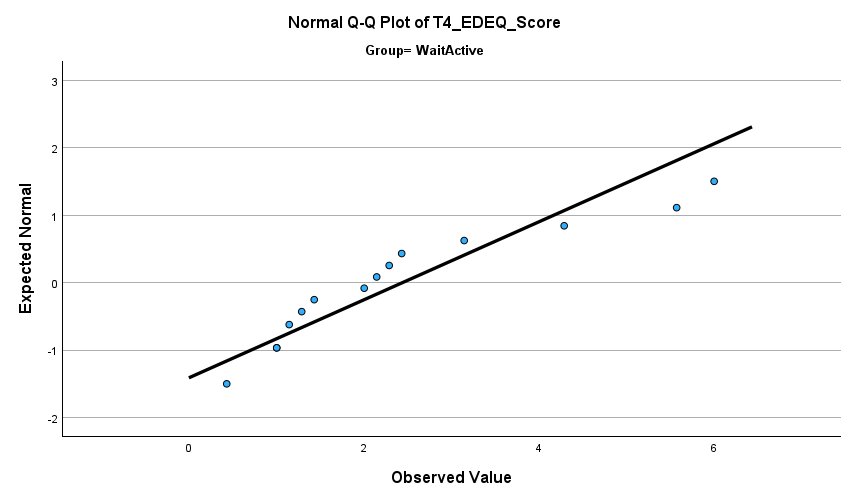


**EDEQ-Drive for Thinness**

***Skewness and Kurtosis***

|  | **Wait-active** | | **Active Wait** | |
| --- | --- | --- | --- | --- |
|  | **Skewness** | **Kurtosis** | **Skewness** | **Kurtosis** |
| **Time point 1** | 0.663 | -0.889 | 0.906 | 0.513 |
| **Time point 2** | 0.495 | -0.860 | 0.811 | 0.256 |
| **Time point 3** | 0.966 | -0.269 | 1.015 | -0.286 |
| **Time point 4** | 0.976 | -0.521 | 0.154 | -1.668 |

***Shapiro-Wilk Test***

|  | **Wait-active** | | **Active Wait** | |
| --- | --- | --- | --- | --- |
|  | **Statistic** | **Significance** | **Statistic** | **Significance** |
| **Time point 1** | 0.880 | 0.057 | 0.919 | 0.210 |
| **Time point 2** | 0.918 | 0.204 | 0.916 | 0.198 |
| **Time point 3** | 0.847 | 0.02 | 0.832 | 0.013 |
| **Time point 4** | 0.814 | 0.008 | 0.878 | 0.054 |


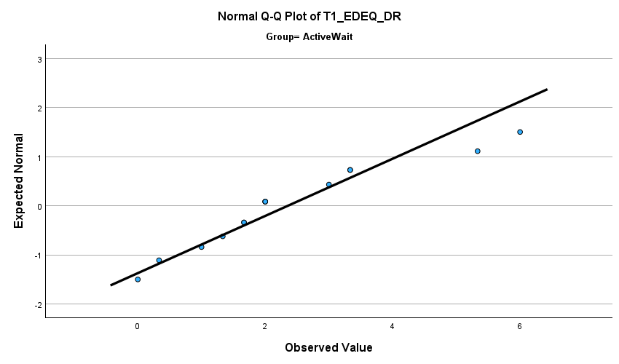

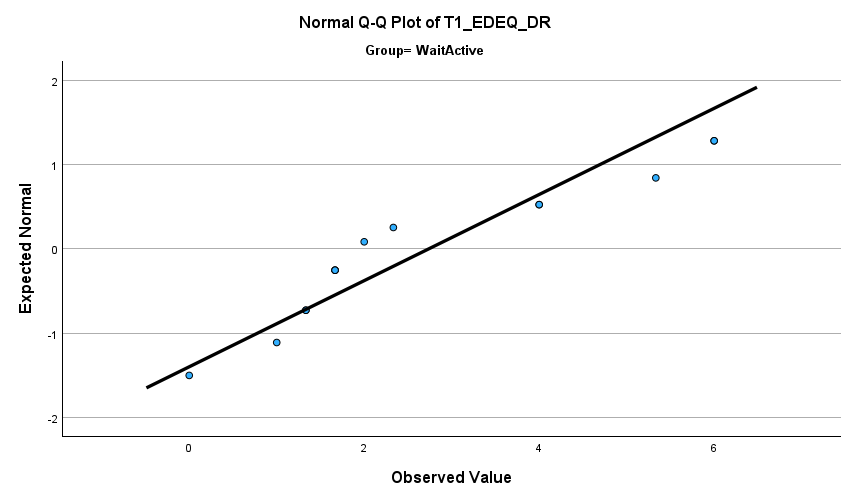


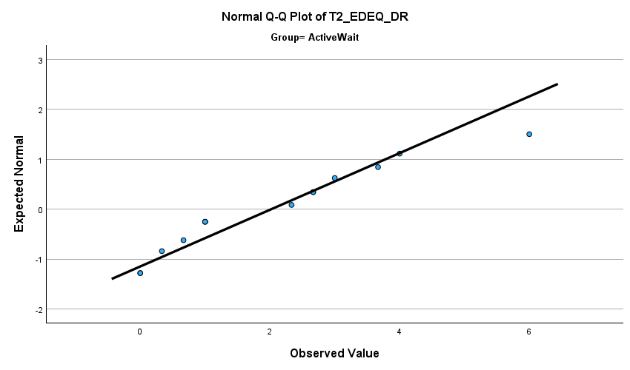

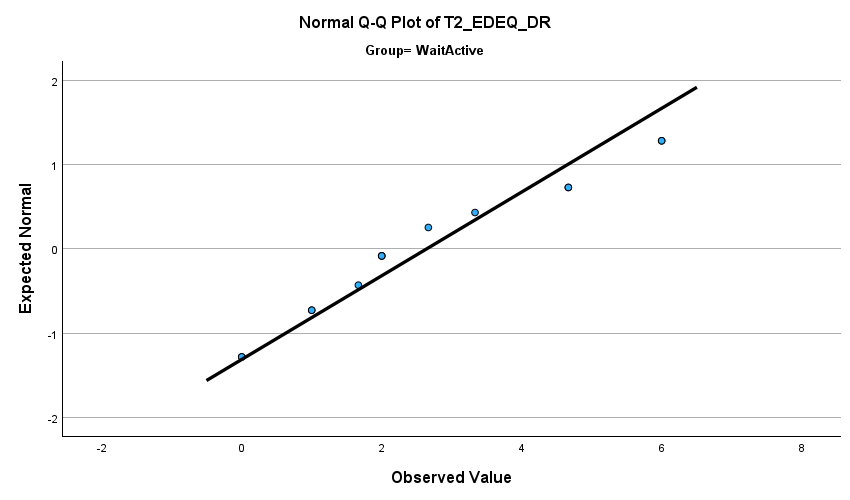


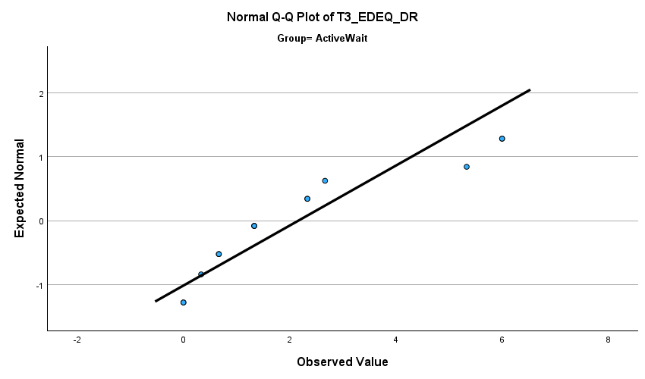

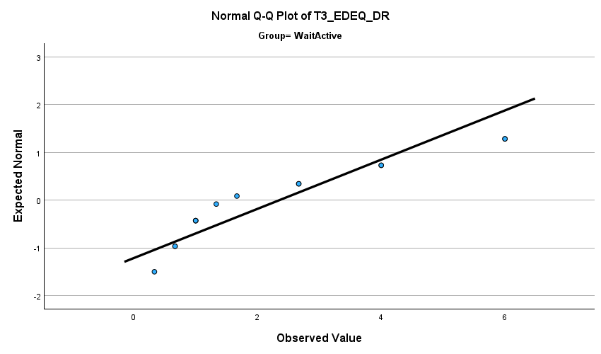


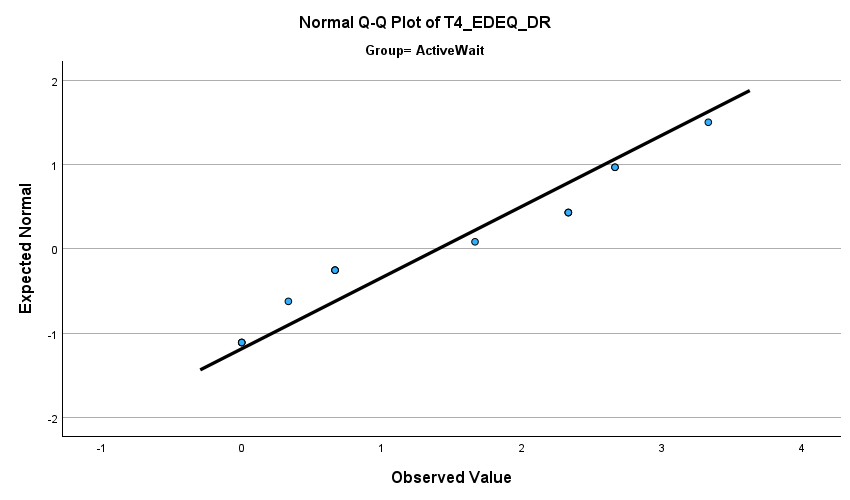

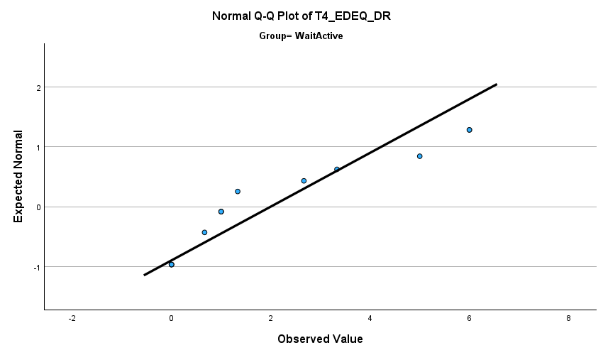


**EDEQ-Shape and Weight Over-evaluation**

***Skewness and Kurtosis***

|  | **Wait-active** | | **Active Wait** | |
| --- | --- | --- | --- | --- |
|  | **Skewness** | **Kurtosis** | **Skewness** | **Kurtosis** |
| **Time point 1** | -0.646 | -0.961 | -0.403 | -0.237 |
| **Time point 2** | -0.256 | -1.084 | 0.433 | -0.235 |
| **Time point 3** | 0.563 | 0.563 | 0.783 | -0.478 |
| **Time point 4** | 0.916 | -0.562 | -0.123 | -0.889 |

***Shapiro-Wilk Test***

|  | **Wait-active** | | **Active Wait** | |
| --- | --- | --- | --- | --- |
|  | **Statistic** | **Significance** | **Statistic** | **Significance** |
| **Time point 1** | 0.932 | 0.324 | 0.875 | 0.05 |
| **Time point 2** | 0.941 | 0.437 | 0.947 | 0.515 |
| **Time point 3** | 0.894 | 0.093 | 0.939 | 0.401 |
| **Time point 4** | 0.950 | 0.559 | 0.833 | 0.013 |


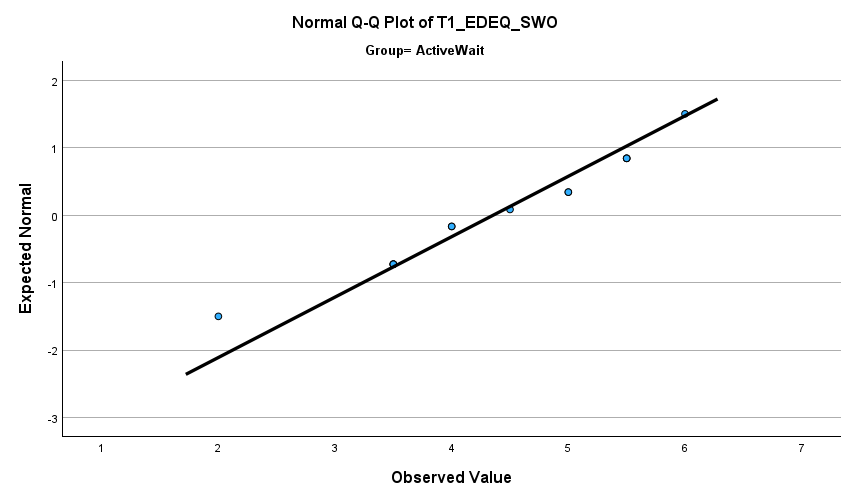

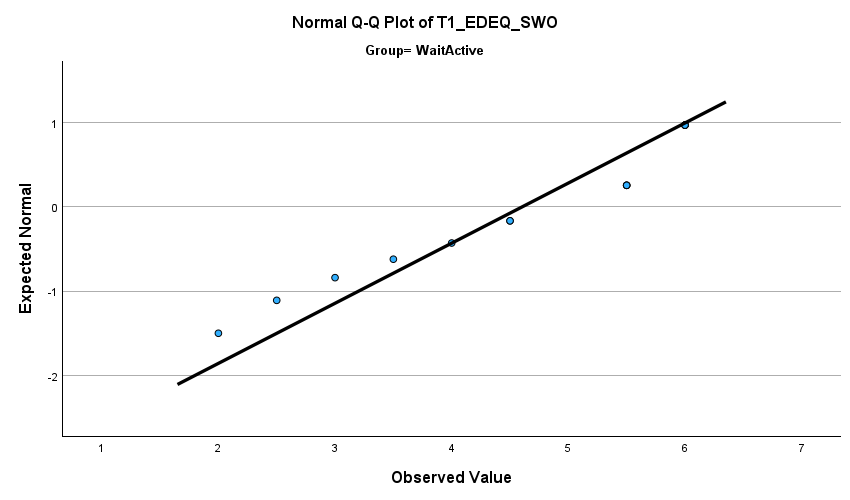


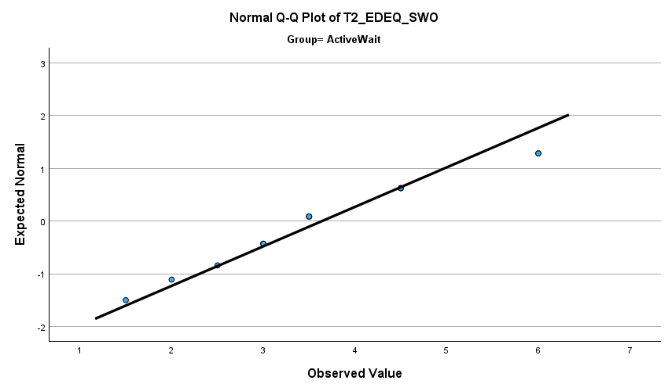

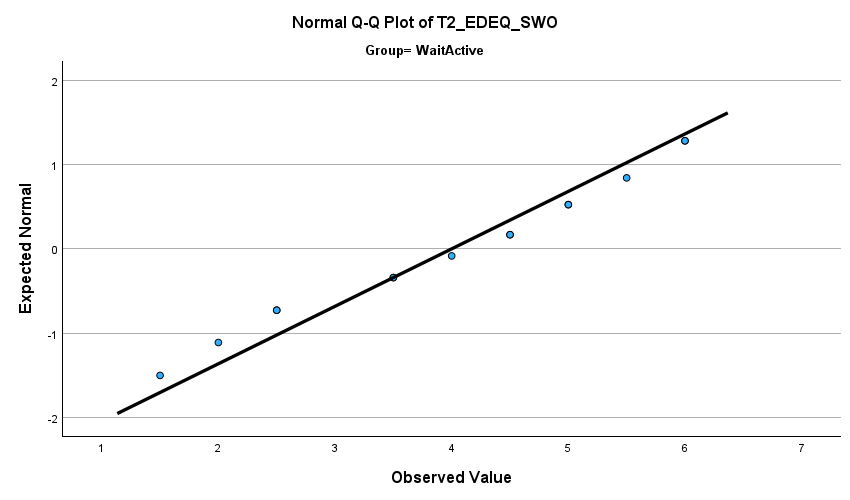


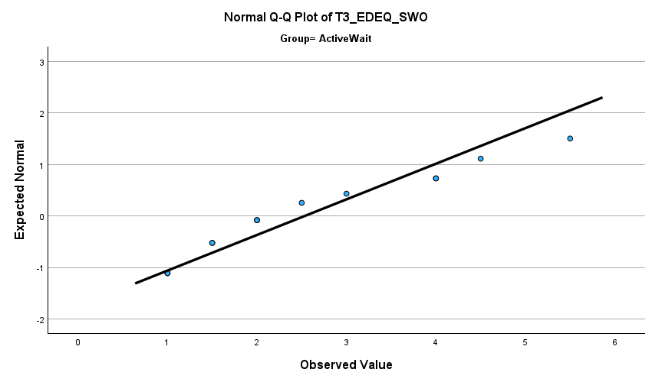

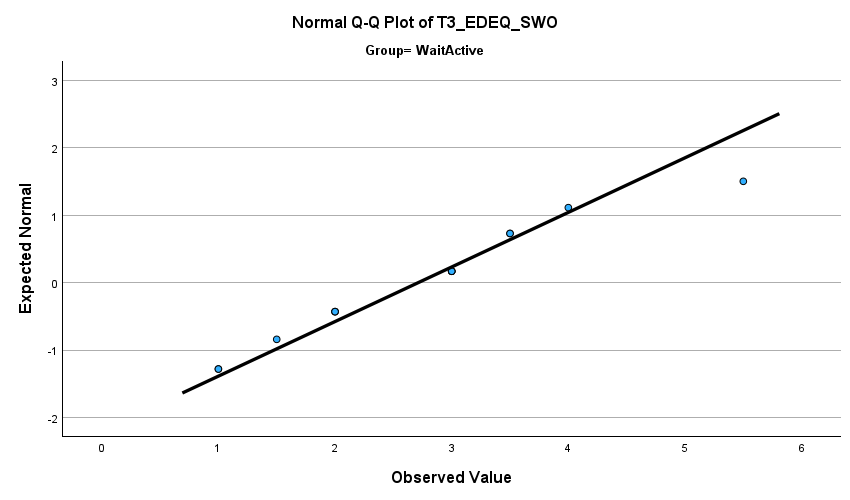


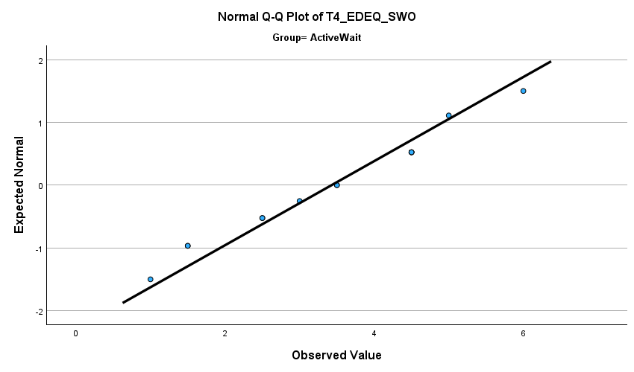

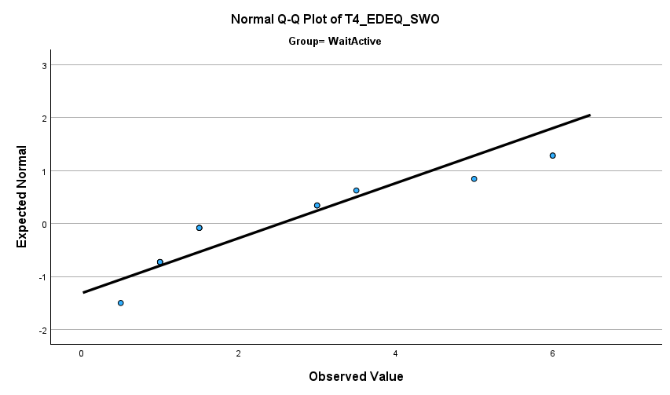


**EDEQ-Body Dissatisfaction**

***Skewness and Kurtosis***

|  | **Wait-active** | | **Active Wait** | |
| --- | --- | --- | --- | --- |
|  | **Skewness** | **Kurtosis** | **Skewness** | **Kurtosis** |
| **Time point 1** | -0.763 | -0.747 | 0.398 | -1.560 |
| **Time point 2** | -0.812 | -0.384 | -0.03 | -0.347 |
| **Time point 3** | 0.307 | -0.438 | 0.03 | 0.155 |
| **Time point 4** | 0.249 | -1.059 | -0.346 | 0.720 |

***Shapiro-Wilk Test***

|  | **Wait-active** | | **Active Wait** | |
| --- | --- | --- | --- | --- |
|  | **Statistic** | **Significance** | **Statistic** | **Significance** |
| **Time point 1** | 0.842 | 0.17 | 0.859 | 0.03 |
| **Time point 2** | 0.959 | 0.711 | 0.893 | 0.09 |
| **Time point 3** | 0.979 | 0.971 | 0.961 | 0.732 |
| **Time point 4** | 0.962 | 0.749 | 0.928 | 0.284 |

**
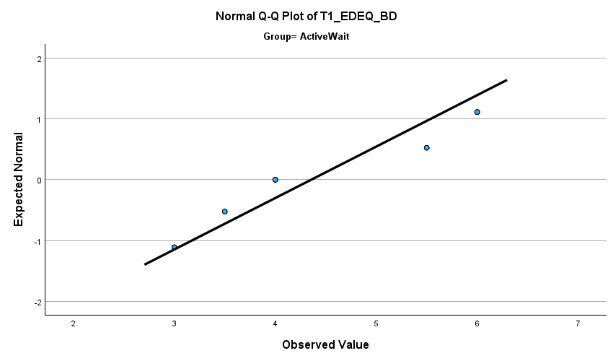

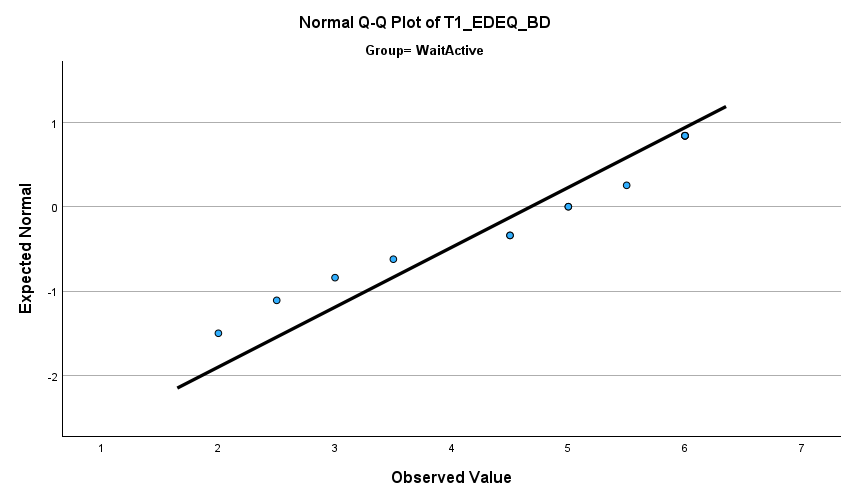
**

**
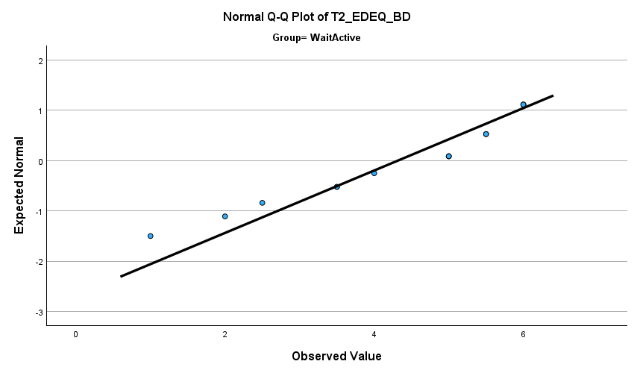

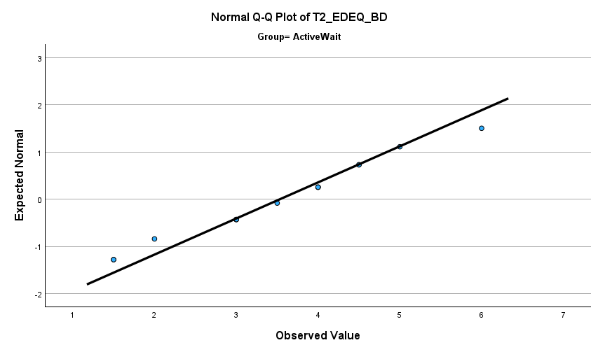
**

**
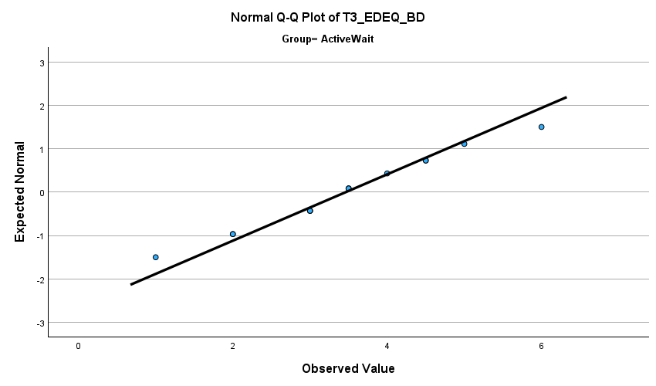

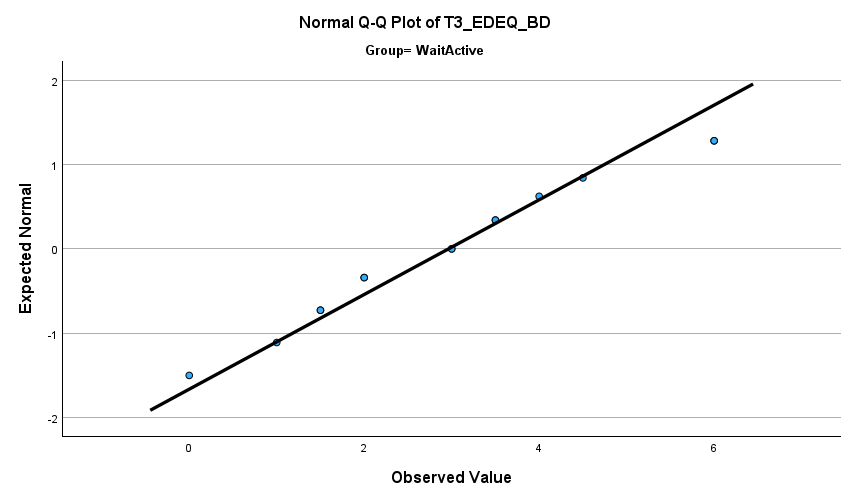
**

**
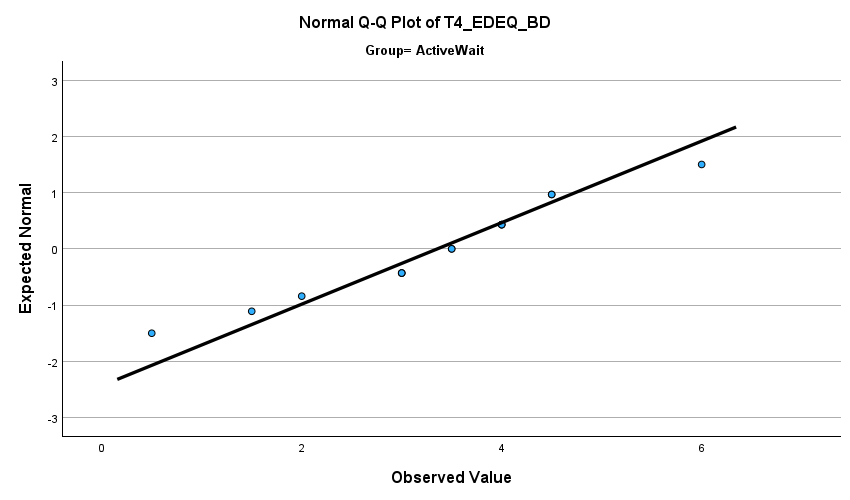

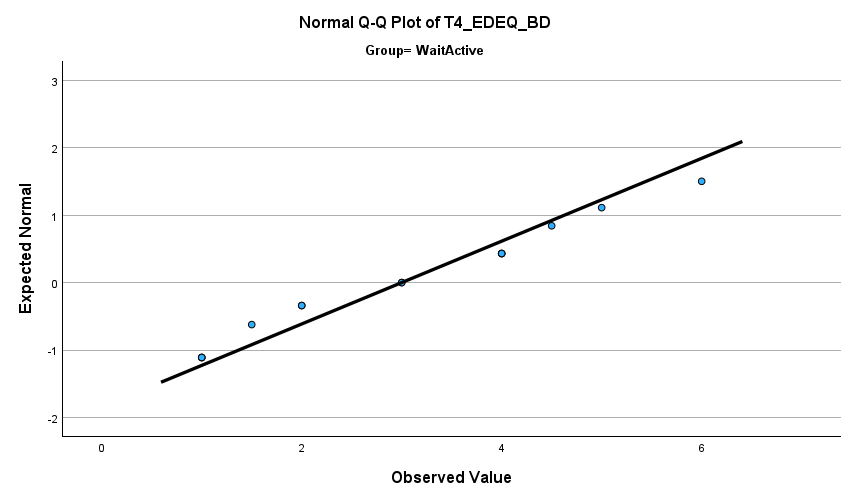
**

**PHQ-Anxiety**

***Skewness and Kurtosis***

|  | **Wait-active** | | **Active Wait** | |
| --- | --- | --- | --- | --- |
|  | **Skewness** | **Kurtosis** | **Skewness** | **Kurtosis** |
| **Time point 1** | -0.479 | -0.781 | 1.276 | 0.835 |
| **Time point 2** | 0.045 | -0.156 | 0.871 | 0.856 |
| **Time point 3** | 0.406 | -0.164 | 0.560 | -0.775 |
| **Time point 4** | -0.093 | -1.362 | 0.438 | -1.302 |

***Shapiro-Wilk Test***

|  | **Wait-active** | | **Active Wait** | |
| --- | --- | --- | --- | --- |
|  | **Statistic** | **Significance** | **Statistic** | **Significance** |
| **Time point 1** | 0.928 | 0.288 | 0.806 | 0.006 |
| **Time point 2** | 0.931 | 0.314 | 0.875 | 0.049 |
| **Time point 3** | 0.920 | 0.222 | 0.889 | 0.077 |
| **Time point 4** | 0.917 | 0.200 | 0.878 | 0.055 |


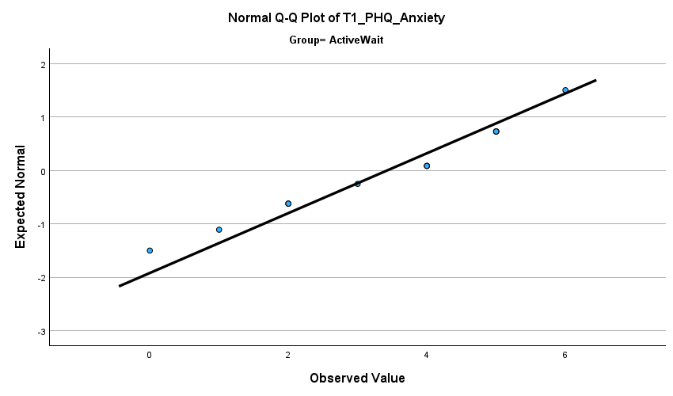

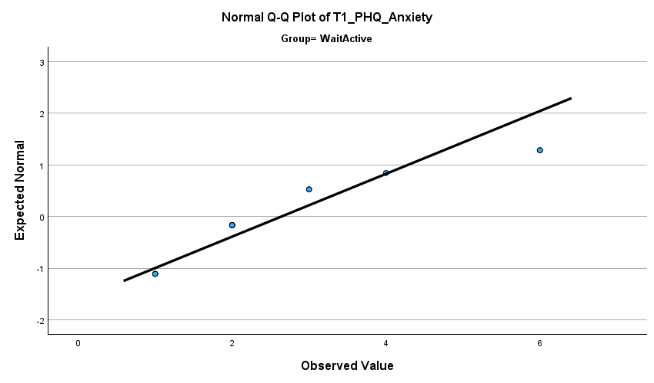


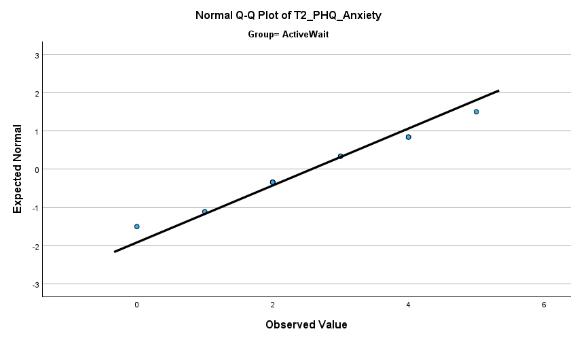

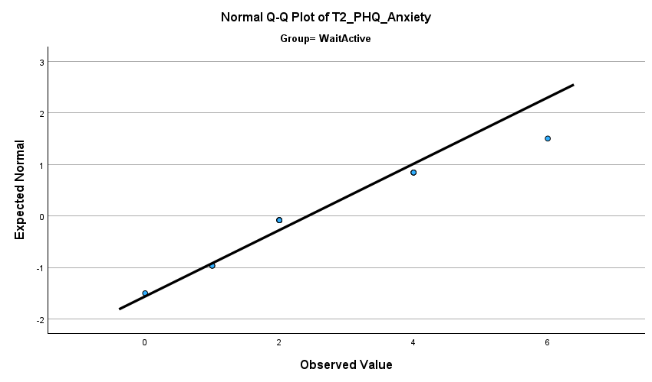


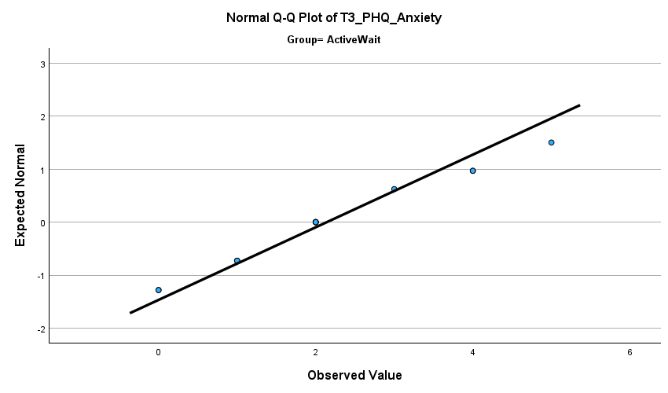
**
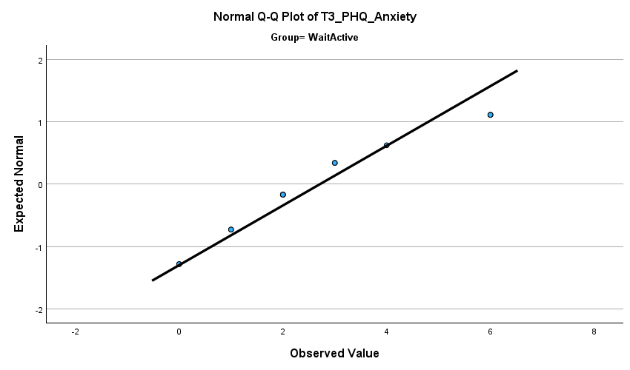
**

**
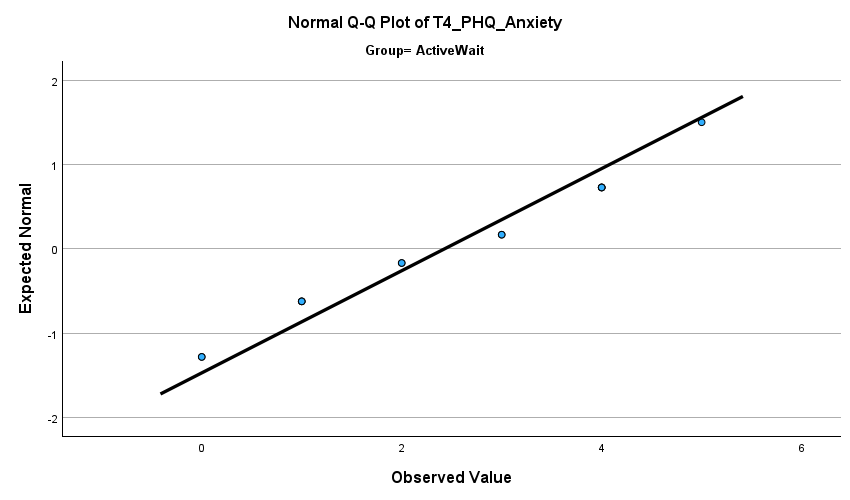

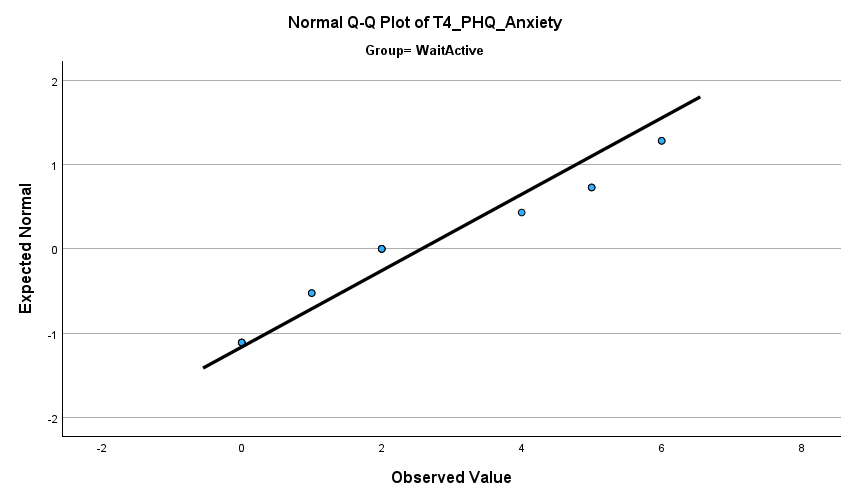
**

**PHQ-Depression**

***Skewness and Kurtosis***

|  | **Wait-active** | | **Active Wait** | |
| --- | --- | --- | --- | --- |
|  | **Skewness** | **Kurtosis** | **Skewness** | **Kurtosis** |
| **Time point 1** | 0.586 | 1.096 | 0.713 | -0.349 |
| **Time point 2** | 1.064 | 0.521 | -0.625 | -1.192 |
| **Time point 3** | 0.511 | -1.142 | 0.321 | -0.633 |
| **Time point 4** | 0.625 | -1.140 | 1.253 | 2.109 |

***Shapiro-Wilk Test***

|  | **Wait-active** | | **Active Wait** | |
| --- | --- | --- | --- | --- |
|  | **Statistic** | **Significance** | **Statistic** | **Significance** |
| **Time point 1** | 0.881 | 0.06 | 0.881 | 0.59 |
| **Time point 2** | 0.831 | 0.013 | 0.767 | 0.002 |
| **Time point 3** | 0.866 | 0.037 | 0.806 | 0.006 |
| **Time point 4** | 0.862 | 0.032 | 0.858 | 0.028 |


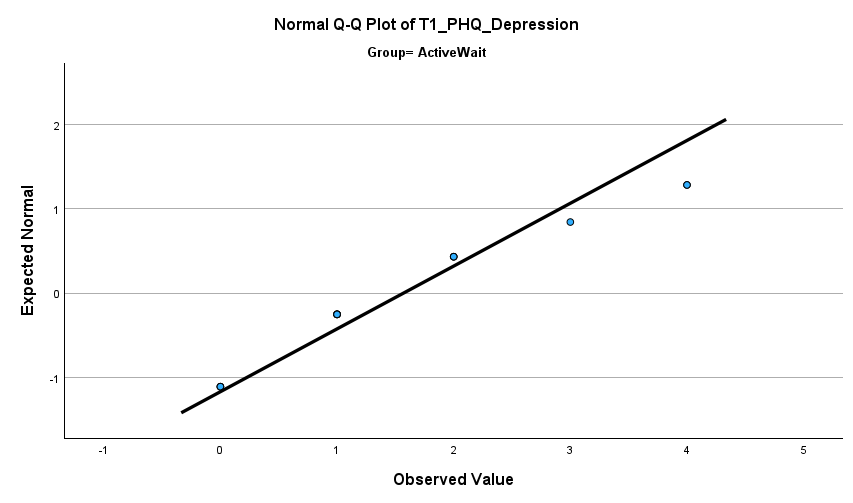

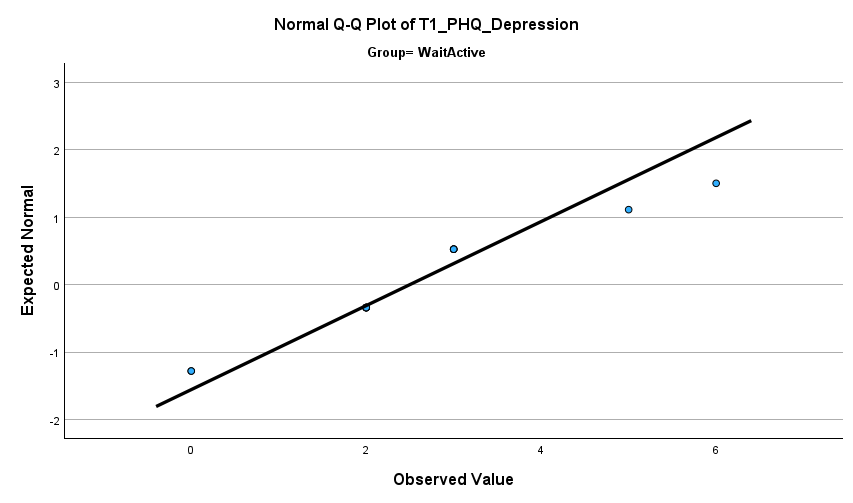

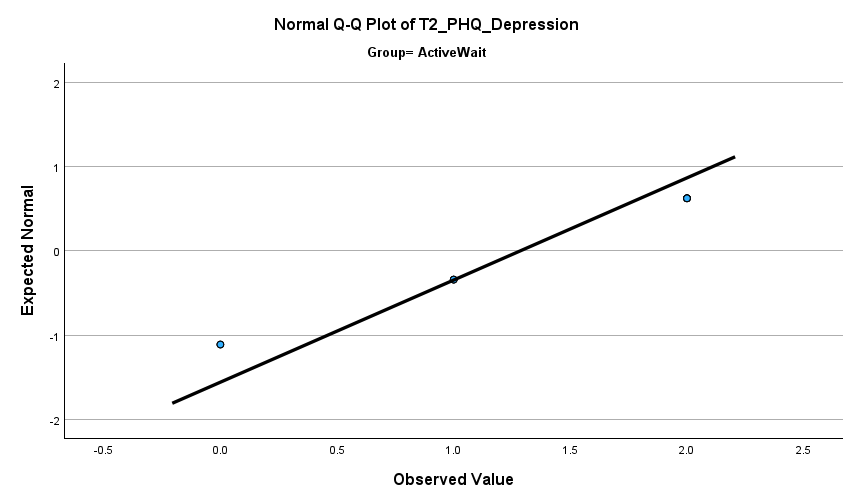

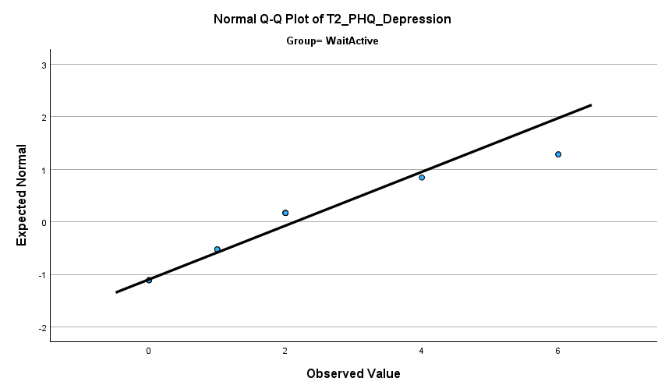


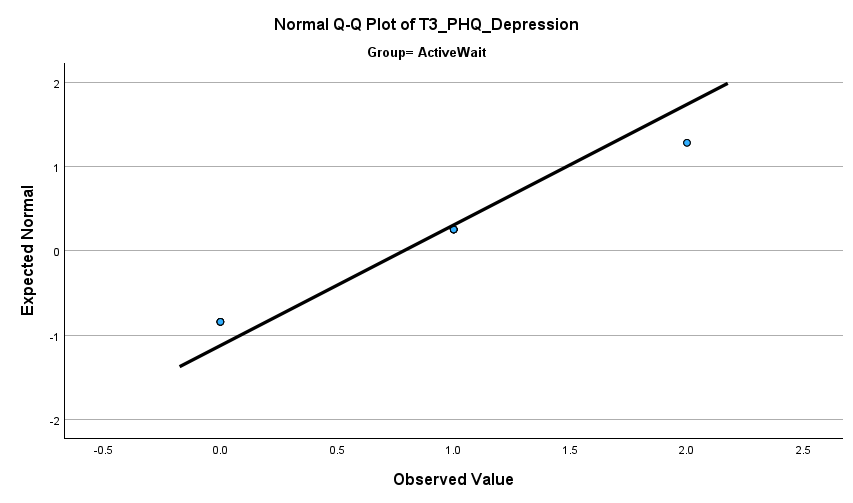

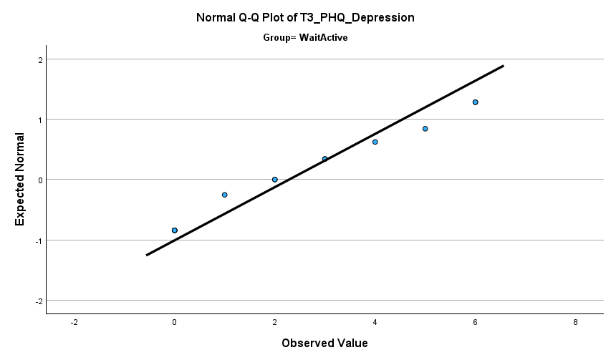

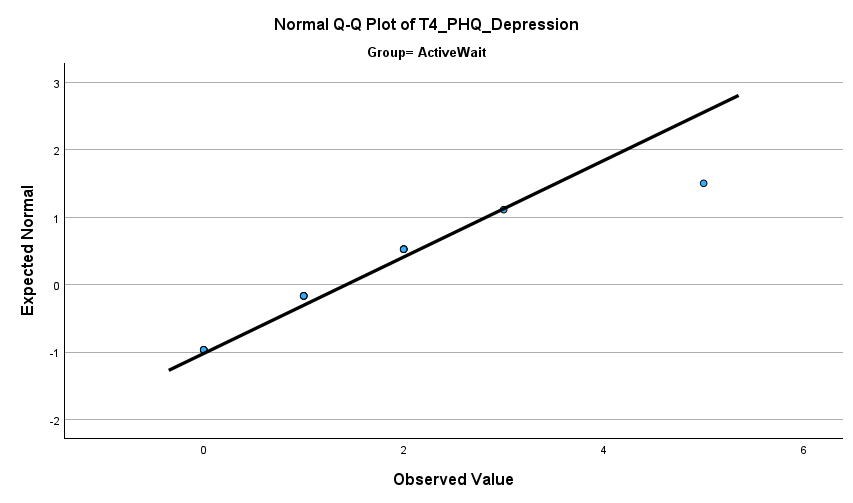

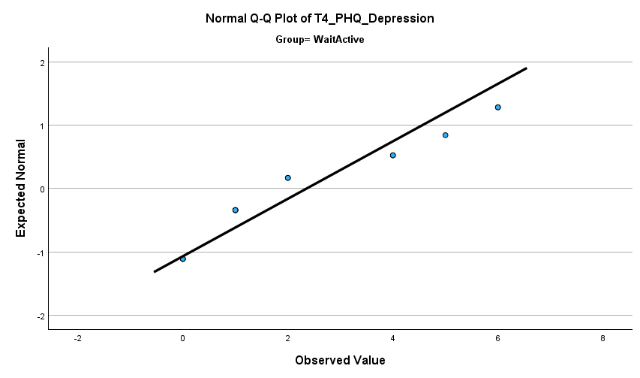

Supplement: Supplementary file 2 — Data S2: Supporting Information. [file EAT-59-371-s001.docx]
